# DynaDom: structure-based prediction of T cell receptor inter-domain and T cell receptor-peptide-MHC (class I) association angles

**DOI:** 10.1186/s12900-016-0071-7

**Published:** 2017-02-02

**Authors:** Thomas Hoffmann, Antoine Marion, Iris Antes

**Affiliations:** Department of Biosciences and Center for Integrated Protein Science Munich, Technische Universität München, Emil-Erlenmeyer-Forum 8, 85354 Freising, Germany

**Keywords:** T-cell recognition, TCR structural modeling, Epitope prediction, Glutamine side chain prediction, Protein domain association angles, Immunoinformatics, Adoptive T-cell therapy, Vaccine design

## Abstract

**Background:**

T cell receptor (TCR) molecules are involved in the adaptive immune response as they distinguish between self- and foreign-peptides, presented in major histocompatibility complex molecules (pMHC). Former studies showed that the association angles of the TCR variable domains (Vα/Vβ) can differ significantly and change upon binding to the pMHC complex. These changes can be described as a rotation of the domains around a general Center of Rotation, characterized by the interaction of two highly conserved glutamine residues.

**Methods:**

We developed a computational method, DynaDom, for the prediction of TCR Vα/Vβ inter-domain and TCR/pMHC orientations in TCRpMHC complexes, which allows predicting the orientation of multiple protein-domains. In addition, we implemented a new approach to predict the correct orientation of the carboxamide endgroups in glutamine and asparagine residues, which can also be used as an external, independent tool.

**Results:**

The approach was evaluated for the remodeling of 75 and 53 experimental structures of TCR and TCRpMHC (class I) complexes, respectively. We show that the DynaDom method predicts the correct orientation of the TCR Vα/Vβ angles in 96 and 89% of the cases, for the poses with the best RMSD and best interaction energy, respectively. For the concurrent prediction of the TCR Vα/Vβ and pMHC orientations, the respective rates reached 74 and 72%. Through an exhaustive analysis, we could show that the pMHC placement can be further improved by a straightforward, yet very time intensive extension of the current approach.

**Conclusions:**

The results obtained in the present remodeling study prove the suitability of our approach for interdomain-angle optimization. In addition, the high prediction rate obtained specifically for the energetically highest ranked poses further demonstrates that our method is a powerful candidate for blind prediction. Therefore it should be well suited as part of any accurate atomistic modeling pipeline for TCRpMHC complexes and potentially other large molecular assemblies.

**Electronic supplementary material:**

The online version of this article (doi:10.1186/s12900-016-0071-7) contains supplementary material, which is available to authorized users.

## Background

**Fig. 1:**
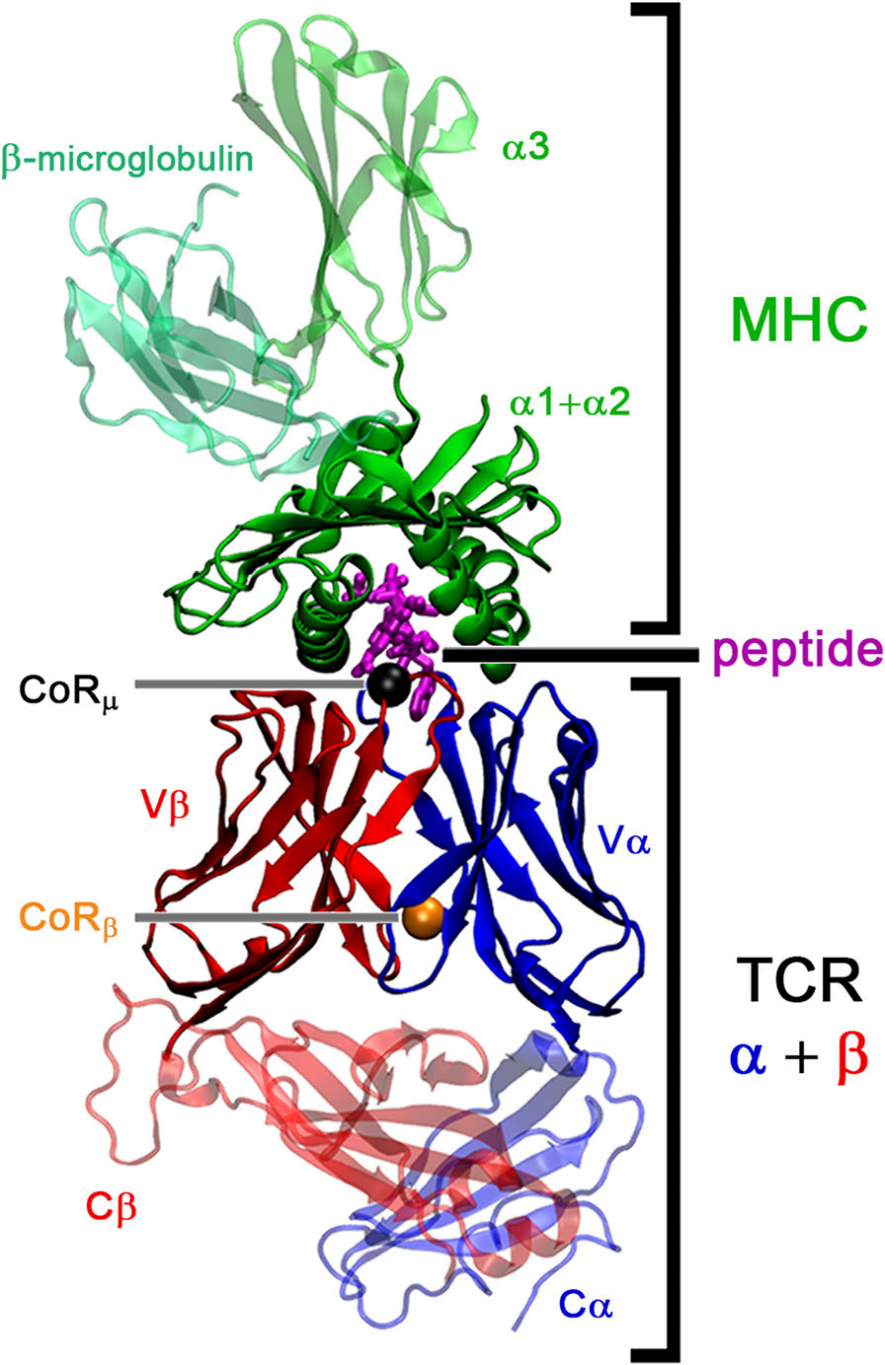
Representation of the TCRpMHC complex (PDB-ID 2bnq). The MHC class I molecule is depicted in *green* (i.e., α1, α2, and α3 chains). The β-microglobulin is colored in *cyan* and the peptide bound to MHC in *magenta*. The two chains of TCR, α and β, are represented in *blue* and *red* colors, respectively. In the present application, the domains shown as transparent are removed from the structure, and only the two variable domains of TCR (i.e., Vα and Vβ), the α1 and α2 chains of MHC as well as the peptide are modeled. In addition, the two centers of rotations CoR_β_ and CoR_μ_, are respectively represented by an *orange* and a *black* colored ball

An early event in the T cell mediated immune response is the recognition of pathogenic peptides contained in major histocompatibility complex (MHC) molecules. The capability of the vertebrate immune system to distinguish between a vast variety of pathogenic- and self-peptides is achieved by a tremendous population of different T cell variants (i.e., in a magnitude estimated from 10^6^ to 10^7^), which differ from each other in the T cell receptor (TCR) [1–3]. Such a diversity results from the combination of two membrane anchored TCR chains (α and β), which are encoded by gene segments joined in a process known as v(d)j recombination [4]. As depicted in Fig. 1, each chain consists of two immunoglobulin-like domains, the variable domain (further referred to as Vα and Vβ) and the constant domain. The v(d)j combination process occurs during the T cell maturation in the thymus, where variable (v) and joining (j) gene segments are combined while nucleotides are randomly introduced within the variable domains (V). In the case of the Vβ domain, an additional short segment is inserted in between the v and j segments, further increasing the TCR diversity (d). The binding interface of the TCR to the peptide-MHC molecule complex (pMHC) is formed by loops named as complementary determining regions (CDR), and each chain of TCR contains three CDRs. While the primary structure of CDR1 and CDR2 loops evolved together with the MHC molecules [5], the sequence of CDR3 loops is determined by the v(d)j recombination and thus exhibits a higher diversity [6].

The number of resolved bound and unbound TCR structures has drastically increased to 200 in the Protein Data Bank [7] during the past few years. Nevertheless, considering the vast variety of TCRs and the high polymorphism of the MHC molecules, the development of reliable structural methods is of crucial importance in order to complement time consuming experimental structural techniques [8]. Such modeling approaches can help in the field of rational TCR design/optimization (e.g.*,* adoptive T cell cancer therapy) [9, 10], in the context of vaccine design [11, 12], and in the development of a consistent theory for T cell signal transduction, which is still not fully understood [13].

Over the past two decades, many theoretical methodologies have been developed and applied to model and predict TCRpMHC interactions.

The main focus in the area has been on the prediction of the MHC/peptide interaction without explicit consideration of the T-cell receptor as the experimental study of MHC-peptide binding has been a very active field since the mid-90s whereas the systematic investigation of the T cell response started about a decade later. In addition, MHC-peptide binding is a necessary prerequisite for the T cell response and thus has by itself already a highly predictive value. Therefore various sequence and structure based prediction tools have been developed of MHC-peptide binding in the past decades [14, 15]. Next to MHC-peptide specific structure-based prediction methods such as EpiDock, PREDEP, pDOCK, DynaPred, or DockTope [16–20], also general molecular docking approaches were applied [21, 22].

The first atomistic model of a TCRpMHC complex was built in 1995 by Almagro et al. using homology modeling and molecular dynamics techniques [23], before the first X-ray structures of a TCR (1tcr [24]) and of a TCRpMHC complex (1ao7 [25]) were solved in 1996. Later, Michielin et al. realized a homology model of the T1 TCR structure bound to the photoreactive PbSC peptide and to the murin K^d^ MHC class I molecule, using the 1ao7 crystal structure of the TCRpMHC complex as a template [8]. The authors applied a methodology combining the MODELLER program with simulated annealing techniques [26], and suggested a rational homology model, which was refined based on previous mutation studies [27]. Further developments of the approach led to the TCRep 3D method [28], which was recently applied in the context of rational TCR design [10]. In addition, Haidar et al. enhanced the affinity of the A6 TCR to TAX:HLA-A2 for about 100-fold using a structure-based model [29]. More recently, Pierce et al. [30] developed an approach based on their scoring function ZAFFI and on the Rosetta interface mutagenesis tool [31] to identify relevant point mutations that could increase the affinity of a TCR to a pMHC complex in the field of therapeutic immunology. The method allowed to optimize the DMF5 TCR to bind the ELAGIGILTV:HLA-A2 complex with a remarkable ~400-fold higher affinity. The same group also developed TCRFlexDock, a method to model a pMHC ligand onto a TCR that takes advantage of the Monte Carlo-based RosettaDock protocol [32, 33]. For a benchmark test set of twenty structures [33], the prediction of near native models was reached in 80% of the cases. The TCRFlexDock method was recently applied to predict models of TCRs bound to MHC like ligands such as CD1 and MR1 [34]. In that work, the authors showed that the use of multiple docking starting positions significantly improves the performance of the prediction.

In order to achieve an accurate molecular model of TCRpMHC complexes, it is necessary to consider several topological aspects of this sophisticated system. First, a precise description on an atomistic level is required, since small alterations in the TCR’s or in the ligand’s sequence can drastically affect the transduced signal [35]. As it was shown in other studies, mutations in the receptor or in the ligand can modify the binding affinity and thus the relative placement of the two units of the complex [36–39].

A second aspect to consider is the variation of the Vα/Vβ inter-domain angle within the TCR, as this is a system specific feature, and as it can adapt upon binding of the pMHC. The analysis of the inter-domain angle between the Vα and Vβ TCR domains as well as its influence on the binding of pMHC was analyzed in several computational studies, which compared broad sets of TCR structures. Notably, by applying the pseudo-dyad method, McBeth et al. suggested that the resulting observed differences between the free and the MHC bound forms of TCRs constitute a feature of the receptor to adapt to different ligands, thus allowing cross reactivity [40]. Dunbar et al. analyzed a non-redundant set of TCRs with the ABangle methodology [41], which describes both the Vα- and the Vβ-orientation in an absolute manner, by considering a torsion angle, four bend angles and one distance as descriptors [42]. In the context of rational TCR-like antibody design, the authors found that antibodies adopt angles comprised in a different range than the one observed for TCRs. In our previous work [43], we analyzed the relative Vα- and Vβ-orientation by reducing the variable domains to cuboids, which served as basis for a distance based clustering. We observed that TCRs belonging to the same clonotype associate in the same angular cluster. Furthermore, we identified a Center of Rotation (further referred to as CoR_β_ and depicted in Fig. 1) and determined its location in the middle of a conserved interaction between two glutamine residues, one in the Vα and one in the Vβ domain. The various inter-domain angles in the evaluation set could be obtained through a rotation around this center. Recent studies, including ours, further emphasized the large range of values that the TCR Vα/Vβ inter-domain angle can adopt [40, 43, 44] and thus its influence on the positioning of the ligand binding CDR loops. These results suggest that next to the orientation of the pMHC ligand with respect to the TCR [24, 25, 36, 45, 46], also the Vα/Vβ inter-domain angle should be explicitly taken in account to assess an accurate homology modeling of TCRpMHC complexes. This last comment is in agreement with recent observations about the dynamics of the TCRpMHC system and the influence of the TCR on the pMHC structure [44, 47]. In addition, it was shown for antibodies that the consideration of the V_H_/V_L_ angles for homology modeling can increase the accuracy considerably [41, 48, 49]. In this context, Dunbar et al. identified key structural parameters, which provide a comprehensive description of the movement of the V_H_ and V_L_ domains with respect to each other [41]. Based on these features and on their respective values in the available antibody structures, Bujotzek et al. trained a predictor for the association of the two domains [48]. The authors further concluded that the consideration of the association angles is crucial for the prediction of highly accurate homology models of antibodies [49].

Along the course of the present study, we pointed out a third topological aspect that can have an impact on the success of TCRpMHC complexes modeling. The Vα/Vβ orientation directly depends on the proper interaction of two specific glutamine residues. During protein structure elucidation by X-ray crystallography, the ambiguous electron densities of nitrogen and oxygen atoms can hamper the correct assignment of these two elements. In the case of asparagine and glutamine residues, this often leads to misassigned atoms in the carboxamide group of the side chain. The detailed investigation of high-resolution structures shows that approximately 20% of these residues are assigned in a wrong flip state, leading to a non-optimal hydrogen bond network [50–53]. The respective orientation of asparagine and glutamine residues has a dramatic impact on most of molecular modeling techniques [53], and should be corrected by considering their direct environment. Due to this significance, several approaches have been developed in order to address this issue. Among those, the most popular ones are HBPLUS (X-PLOR package) [52], NETWORK (WHAT IF package) [53], Reduce (MolProbity package) [50, 54, 55], NQ Flipper [51, 56, 57], the Independent Cluster Decomposition Algorithm (ICDA) [58], Protonate 3D [59], Protoss [60, 61], and the Computational Titration method [62].

Despite the great improvements in TCRpMHC complex modeling achieved during the past decades, some of the critical aspects described above are still not taken into account. To the best of our knowledge, none of the currently available methodologies explicitly include the adaption of the Vα/Vβ inter-domain angles, although these have a direct impact on the disposition of the CDR loops, and as a consequence, on the contact between the TCR and the pMHC ligand.

**Fig. 2:**
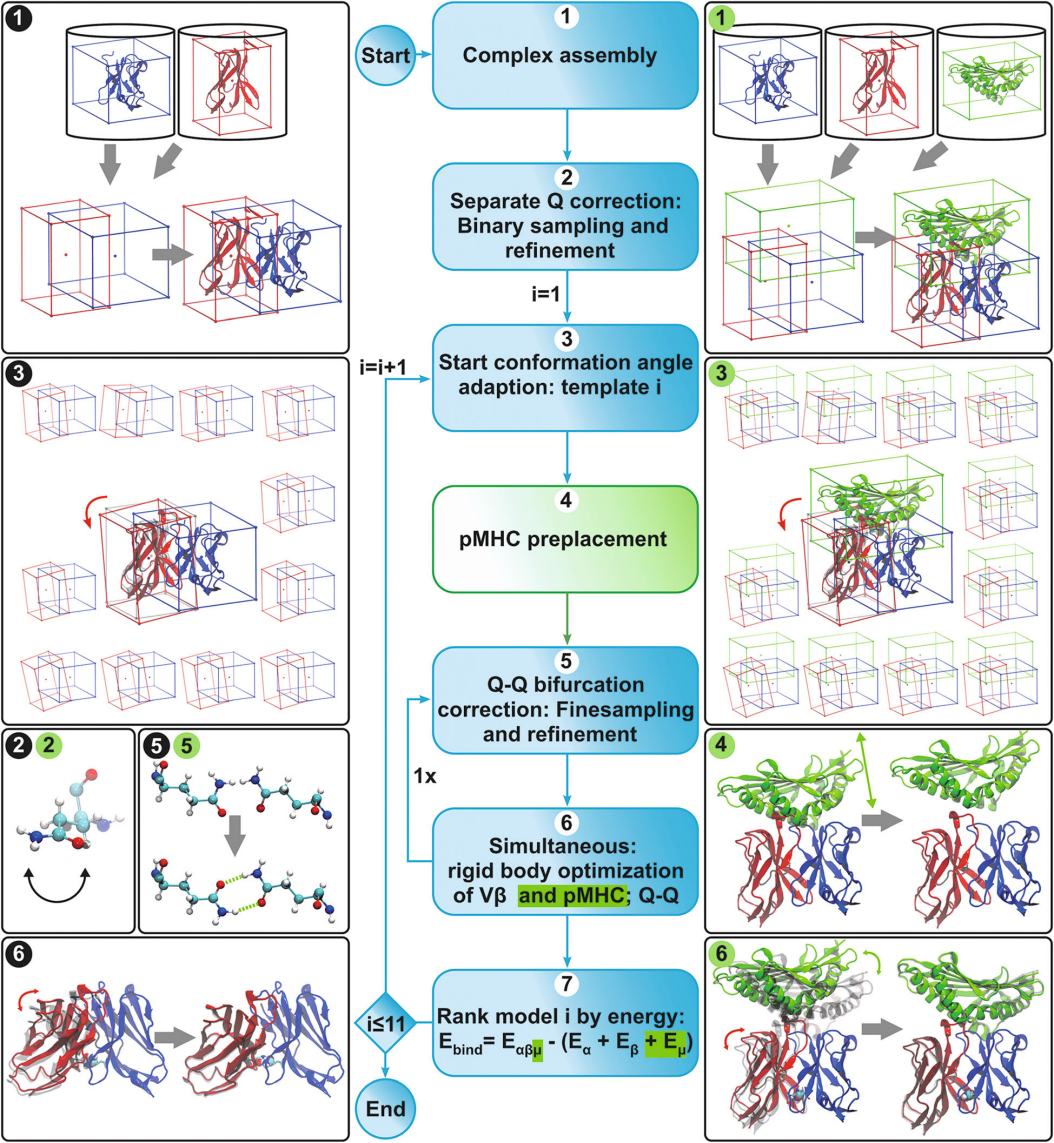
TCR and TCRpMHC complexes modeling pipeline. Center column: standard pipeline (see Methods) for the remodeling of the TCR Vα/Vβ association angles and for the pMHC positioning with respect to the TCR. *Blue* highlighted steps are performed in both modeling pipelines: only Vβ and combined Vβ/pMHC placement. *Green* highlighted steps are performed only if the pMHC is included in the remodeling process. The left and the right columns illustrate the individual steps of the pipeline. Steps with numbers circled in *black*: TCR Vα/Vβ association angle modeling pipeline, steps with numbers circled in *green*: combined Vβ/pMHC modeling pipeline. Steps 3 to 7 are performed for each of the 11 starting conformations. The protein domains represented in *blue*, *red*, and *green* color correspond to the Vα, Vβ, and pMHC units, respectively. In step 1 (both for TCR and TCRpMHC modeling), the different protein domains are described by unified cuboids and assembled. The illustration of steps 2 and 5 show the Q-flip correction/optimization. At step 2, each glutamine residue is optimized with respect to its direct environment only (only the corresponding variable domain is accounted for). Whereas in step 5, the two glutamine residues are optimized simultaneously, thus accounting for the whole TCR environment. In step 4 (only for TCRpMHC modeling), the pMHC unit is pre-placed, translated away from the TCR and optimized with respect to the fixed TCR variable domains. At step 6 (both for TCR and TCRpMHC modeling), the position of all cuboids as well as the orientation of the glutamine residues are optimized concurrently. The latter illustrations show the structure before and after optimization, with the target crystal structure depicted in *gray*

In what follows, we present a new method, DynaDom, for the prediction of TCR Vα/Vβ inter-domain and TCR/pMHC association angles. We implemented our approach into the DynaCell suite [63], a general force-field-based molecular modeling program developed in our group. Our new method uses an extendable multidimensional rigid body optimization approach based on the work by Mirzaei et al. [64]. The implementation is specifically designed in a way that allows for an arbitrary definition of rigid bodies and for the inclusion of local flexibility on different levels (e.g., from the domain to the residue level) into the modeling pipeline (Fig. 2). As a first application, we evaluate here the DynaDom method for the remodeling of a large set of TCR and TCRpMHC complexes. This evaluation intends to determine the general capability of a rotation-based algorithm and the relevance of our CoR-concept [43] for the successful prediction of association angles. Notably, we demonstrate here that it is possible to distinguish between correct and wrong models by solely using the force-field-based interaction energy computed between the different units of the complex. This is indeed very promising for future blind homology modeling of TCRpMHC complexes and others, especially if a sufficient amount of experimental data is not available for the training of an application-specific, knowledge–based scoring function.

## Methods

The new DynaDom prediction method presented here is based on the concepts developed for our previous analysis of the structural features of TCRpMHC complexes [43] and uses the same theoretical framework as defined therein. In ref. [43], we performed a comprehensive and systematic analysis of the Vα/Vβ inter-domain angles in a set of 85 structures, by representing each domain as a unified cuboid (for a brief summary of the methodology, see Additional file 1: Text S1A). The main results of that former work can be summarized as follows: i) we showed that the TCR complexes of the analysis set can be grouped into six structural clusters, by solely using the Vα/Vβ inter-domain angle as a descriptor; ii) we identified a conserved center of rotation that determines the orientation of the Vβ domain with respect to Vα (further referred to as CoR_β_); iii) we pointed out that this center of rotation (CoR_β_) is characterized by the interaction of two highly conserved glutamine residues (Q; one per variable domain), forming a stable hydrogen bond linkage between Vα and Vβ.

In the present work, we intend to translate this structural knowledge gained in ref. [43] into a computational pipeline to model TCR and TCRpMHC complexes. We hereafter detail our strategy by first describing the general concepts of DynaDom and the extension of the center of rotation concept to the case of TCRpMHC complexes. Then, we describe the theoretical framework of our rigid body optimization algorithm and give a detailed description of the overall prediction pipeline. Finally, we define the particular data set used in the present test application of DynaDom for the remodeling of TCR and TCRpMHC complexes.

### General concepts of DynaDom

The DynaDom modeling approach is based on the unified cuboid description of a given molecular assembly by assigning one cuboid to each structural domain, as applied to the TCR Vα and Vβ domains in ref. [43]. In the case of TCRpMHC complexes the Vα, Vβ, and pMHC units are represented by three independent cuboids. To further reduce the dimensionality of the problem, we use the Vα domain as internal, fixed coordinate frame (see ref. [43], Fig. 2, and Text S1.A of Additional file 1 for details). In this frame, the placement of the Vβ and pMHC units can be simply described by a series of translation and rotation operations around a given point of the cuboid. For the placement of Vβ with respect to Vα, we here chose this point as the previously identified center of rotation, CoR_β_ (Fig. 1). In a similar manner, we define here a center of rotation for the placement of pMHC units, which we shall refer to as CoR_μ_.

The binding of the pMHC ligands onto TCRs has been suggested to occur in a generally diagonal mode [46], based on the analysis of the early structures of this complex [24, 25, 36]. More recently, Rudolph et al. introduced a general unified method to measure the binding angle of TCRs with respect to their ligands and determined the angular range of 24 complexes [45]. The latter method, based on a general rotational axis, is however too general to describe all the transformations of a pMHC complex in a three-dimensional space. We thus adapted our previously introduced cuboid method [43] and measured the three Euler angle components of the TCR/pMHC orientation. Equivalently to the determination of CoR_β_ (see Additional file 1: Text S1A for details), we define here a center of rotation for the orientation of the pMHC cuboid relative to the Vα domain (CoR_μ_; Fig. 1). Unlike CoR_β_, CoR_μ_ does not correspond any conserved residue and lies at the center of the peptide binding groove of the pMHC complex. As CoR_β_ and CoR_μ_ are solely defined by the Vβ and pMHC coordinates, we further use their location as rotational centers for the rigid body optimization. These locations will be named as CoR_β_- and CoR_μ_-based rotational centers, respectively.

Our strategy for the modeling of TCRpMHC complexes intuitively resembles the plausible biological process: i.e., first the association of the two TCR variable domains, then the approach of the pMHC complex towards the TCR CDR loops (Fig. 2). We thus assume here that the general orientation of the TCR variable domains is determined prior to the binding of the pMHC ligand, and then further adjusts upon binding. As a consequence, comprehensive sampling of possible Vα/Vβ orientations is crucial and can determine the success of the modeling attempt. Based on our former analysis [43], we define here 11 starting orientations for the association of the two TCR variable domains. Such number of different initial conditions is intended to cover the large range of Vα/Vβ inter-domain angles and to avoid artificial local minima. It also increases the probability that at least one of the obtained Vα:Vβ complexes is close enough to the final bound conformation such that it can effectively bind the pMHC ligand (for details about the choice of the 11 starting orientations see Additional file 1: Text S1B).

To determine the starting orientation of the pMHC cuboid, we analyzed the Vα/pMHC angles associated with all structures considered in our set (see subsection Structural data sets). The crystal structure 3e3q [65] showed the lowest angular deviation with respect to the others and was chosen as reference structure. Based on the normal vector to the plane defined by the MHC β-sheet backbone atoms of 3e3q we derived a translation axis for the pMHC.

The 11 starting structures for the TCRpMHC modeling pipeline are obtained such that for each of the 11 starting Vα/Vβ orientations, the pMHC ligand is placed in a general position (based on the pMHC orientation in 3e3q) and afterwards translated away from the TCR along the 3e3q-based translation axis. For each of these structures one cuboid is defined around each domain, i.e. Vα, Vβ, and pMHC.

The relative position of these cuboids is then optimized iteratively along a succession of operations defined by our pipeline algorithm, for each of the 11 starting conformations of a given complex (Fig. 2). Our pipeline is built in a modular manner, such that beside the interaction between the different subunits, it is possible to explicitly model the flexibility of some relevant parts of the molecular system. As we detailed in the Introduction, the center of rotation between Vα and Vβ (CoR_β_) is characterized by the specific interaction of two glutamine residues (Q). However, because of the ambiguous character of the Q side chain, the assignment of the atoms (Q-flip state) in crystal structures often happens to be wrong. Furthermore, the central location of these two residues in the complex makes them particularly sensitive to variations in the Vα/Vβ inter-domain angles. For these reasons, our pipeline also includes a Q-flip correction/optimization module, which is applied alongside with the general optimization of the whole complex.

For the modeling of one TCRpMHC complex, our pipeline algorithm thus results in a total of 11 structures, each of them originating from the corresponding starting orientation of the TCR variable domains. These structures are optimized and finally ranked according to their interaction energy.

### Rigid body optimization

We implemented our method within the DynaCell suite of programs [63] using a rigid body energy minimization approach based on the work by Mirzaei et al. [64] together with the Broyden-Fletcher-Goldfarb-Shanno (BFGS) algorithm as implemented in the GNU scientific library (libGSL; version 1.15 double) [66]. Details about the applied settings of the algorithm and a discussion about its convergence are presented in Additional file 1: Text S1C. Mirzaei et al. introduced the original algorithm focusing on the RBEM problem for molecular docking [64]. The approach is specifically designed for an efficient rotation of the rigid bodies around a center of rotation and is particularly well suited for our application. However, the original method only allows for a simultaneous optimization of the relative position of only two rigid bodies. We therefore extended it such that the simultaneous optimization of the orientation of an arbitrary number of rigid bodies is possible.

We implemented the method in a generalized, modular way, allowing for the individual design of application specific optimization pipelines, based on a given combination of the different functions during runtime. Each pipeline step consists in the assembly of sub-process operators (SOs), which evaluate an objective function and the corresponding gradient to further perform the resulting coordinates transformations. Each SO manipulates the coordinates of a subset of atoms and calculates the value of the objective function within a given environment (i.e., including the whole system or only part of it). So far, we implemented three different families of SOs. We shall briefly describe them below, while a more detailed presentation can be found as Supporting Information (Additional file 2: Text S2).

The first family of SOs consists of the basic operators for the rigid body rotation and translation. The objective function of these operators is computed from the non-bonded interactions between the rigid bodies of interest (not the intra-cuboid interactions). The operators modify the three parameters for the rotation and one for the translation of the rigid body, either simultaneously or independently.

Next to these general operators, we implemented a specific carboxamide group rotation operator. This operator is valid for both asparagine and glutamine residues. It only modifies one parameter, namely the dihedral angle that defines the orientation of the side chain’s carboxamide group. The rotation axis is set along the carbon-carbon bond next to this functional group (i.e., Cβ-Cγ and Cγ-Cδ for an asparagine and a glutamine residue, respectively). The objective function accounts for all bonded energy terms within the corresponding side chain and non-bonded energies within the system (i.e., including the intra-cuboid interactions). This sub-process operator can be used within our prediction pipeline algorithm or independently, and we shall refer to as Q-flip correction tool in the following.

Finally, we implemented a rigid body position restraint operator to prevent unrealistically large translational motions and hence to avoid irrelevant conformations. The objective function in this case consists of a harmonic potential applied on the distance between a given reference and a mobile point. The harmonic penalty is applied if the distance is greater than the defined threshold. For the present modeling of TCRpMHC complexes, CoR_β_ and CoR_μ_ are used to restrain the positions of the Vβ and pMHC cuboids, respectively. The threshold values are set to 7.5 Å for Vβ and to 13.0 Å for pMHC.

### TCRpMHC prediction pipeline

The standard modeling pipeline for the prediction of the Vβ orientation in Vα/Vβ complexes of TCRs as well as the orientation of both Vβ and pMHC in TCRpMHC complexes is summarized in the central panel of Fig. 2. The illustration of the steps (left and right panels of the figure) applied during the modeling of the TCR alone are shown in black circles, while the steps used for the modeling of the TCRpMHC complexes are circled in green color. In addition, an animation of the modeling process is available as Supporting Information (Additional file 3: Movie S1). We hereafter provide a detailed description of this pipeline for the modeling of one given TCRpMHC complex, based on the steps depicted in Fig. 2.


Additional file 3: Movie S1. Example for the prediction pipeline: Remodeling of the structure with the PDB-ID 1ao7. (MOV 2.8 mb)


A modeling attempt starts with the representation of each subunit (Vα, Vβ, and pMHC) as a cuboid and their placement in the reference coordinate frame (Fig. 2, step 1: complex assembly).

After this initial assembly, the Q-flip state of the central glutamine residues located at the CoR_β_ is corrected independently in each of the two TCR variable domains, by accounting only for the interactions within the corresponding domain, Vα or Vβ (Fig. 2, step 2: separate Q correction). At that stage, we only intend to correct the possibly wrong assignment of the Q-flip state in the crystal structure. The interaction with the Q in the opposite domain is not yet considered, as the correct Q-Q assembly is crucially dependent on the final Vα/Vβ association angle, which is still unknown at this stage of modeling. As a consequence, only two orientations are considered, the original one and a rotation of 180°. The orientation presenting the lowest energy is selected and further refined by performing 30 steps of BFGS energy minimization. As here only one parameter needs to be optimized, the optimization process is straightforward and 30 steps are sufficient to reach convergence (for a more detailed discussion of the chosen BFGS settings see Additional file 1: Text S1C).

Based on these two preparation steps, 11 starting orientations of the TCR variable domains are constructed (Fig. 2, step 3: conformational angle adaption) and the pMHC ligand is placed in a general position and translated away from the TCR (Fig. 2, step 4: pMHC pre-placement). Details about this step are discussed in the “General concepts of DynaDom” sub-section and in the Additional file 1: Text S1A/B. The position of the pMHC is pre-optimized with respect to the fixed Vα and Vβ domains, for a maximum of 150 BFGS steps. This step intends to mimic an approach of the pMHC from far towards an already formed TCR assembly. As the position of only one rigid body is optimized here, most of the optimizations converge in less than 150 steps. For the few optimizations that do not meet the BFGS convergence criteria, the energy still drops in few steps and reaches a stable plateau (for a more detailed discussion of the chosen BFGS settings see Additional file 1: Text S1C). As this step constitutes a preparation for the main optimization (step 6), these structures are considered as converged and the modeling pipeline proceeds.

Next, the orientation of the central Q residues is explicitly sampled in the context of the whole TCR assembly (i.e., accounting for the intra- and inter-subsystems interactions) with a fine step of 18°, leading to 400 different orientations (Fig. 2, step 5: Q-Q bifurcation correction). The orientation with the lowest energy is then selected and further minimized for a maximum of 30 steps and used in the next step. This ensures a proper orientation of the Q residues with respect to each other for the current Vα/Vβ orientation. Here again, considering the straightforward parameter space to be optimized and the explicit sampling performed ahead with a fine angle increment, 30 steps are sufficient to reach convergence.

The core step of the DynaDom algorithm takes place after these preparation steps. At this stage, the position of Vβ and pMHC as well as the orientation of the two glutamine residues are concurrently optimized (Fig. 2., step 6: Simultaneous rigid body optimization). The minimization is first conduced for a preliminary 50 steps. Then step 5 is repeated to ensure optimal Q-Q placement and interaction at the center of rotation CoR_β_ with respect to the adjusted global conformation of the complex. Step 6 is finally repeated for a maximum of 2950 steps. Here again, most of the minimizations converge in a few hundred steps. The rare cases in which the BFGS algorithm did not converge were systematically analyzed, showing that in each case, the energy strongly decreases in a few steps and oscillates around a minimum value (see Additional file 1: Text S1C for a detailed discussion). Therefore, these rare cases were also considered as converged in the present work.

Finally, the quality of the current model (i.e., originating from the *i* ^*th*^ starting conformation out of 11 in the present application) is evaluated by computing the complex binding energy (*E*_*i*,*bind*_) as:

*E*_*i*,*bind*_ = *E*_*i*, *complex*_ − (*E*_*α*_ + *E*_*β*_ + *E*_*μ*_),

where *E*_*i*, *complex*_ is the total energy of the complex and *E*_*α*_, *E*_*β*_, and *E*_*μ*_ are the energy terms of the individual complex components Vα, Vβ, and pMHC (notice that these last quantities are constant for each prediction run and are thus computed only once). The energy is evaluated using the OPLS-AA force field [67, 68]. As the current application is a remodeling attempt, we additionally computed the all-atom positional root mean square deviation (RMSD) with respect to the crystal structure for each of the 11 final models.

The ranking of the 11 final models is performed according to their binding energy. For the current remodeling application, we define an energy criterion to assess the success of the remodeling attempt as C_E_. The energy criterion is fulfilled if the model having the best binding energy also bears an RMSD lower than 2 Å with respect to the original crystal structure. To gain more insight into the performance of our DynaDom method, we define a second success criterion based on the RMSD, C_R,_ which allows us to evaluate the performance of the structural modeling by assessing the structural deviation of the model from the corresponding experimental structure. This structural criterion is fulfilled if at least one of the 11 final models has an RMSD lower than 2 Å with respect to the original crystal structure.

### Structural data sets

We selected 75 biological units (BUs) originating from 48 different crystal structures contained in the set that we previously analyzed in ref. [43].

In that study, we observed that the different BUs within a given crystal structure can slightly differ from each other (RMSD < 1 Å), especially in the exact location of side chain atoms. This is presumably due to the relatively high intrinsic flexibility of the complexes or the limited resolution in some of the structures (differs from 1.5 to 3.5 Å). To evaluate the robustness of our method and its capability to tackle such inaccuracies, we included all BUs in our two main datasets. The inclusion of all BUs also results in a larger data set and, as no training of a scoring function is performed (DynaDom is a force-field based approach as described in the previous subsections), introduces no bias to the method itself. In addition, the current datasets only contain structures in which all atoms that are involved in the modeling process were experimentally resolved. Although these atoms or residues could be easily modeled, this would potentially introduce a bias in the set, which we prefer to avoid here. As summarized in Table 1, the TCRpMHC crystal structures selected for this work belong to two different species (i.e., 17 murine and 31 human) and 22 different TCR clono-types (mutations not accounted). The coordinates of each structure were aligned with respect to the conserved residues of the Vα domain, as described in ref. [43] and in the previous subsections. The TCR constant domains, the MHC α_3_ domain, and the β-microglobulin were systematically removed from the structures as well as all non-protein atoms (the discarded domains are represented with transparent colors in Fig. 1). Hydrogen atoms were added and topologies were created for the OPLS-AA force field [67, 68] using the pdb2gmx tool (Version 4.5.6) [69].

**Table 1:** Description of the structural dataset DS_T_ and the subset DS_C_

PDB	DS^a^	TCR-Name	S^b^	L^c^	R^d^
1ao7	T/C	A6	H	I	[25]
1fo0	T/C	BM3.3	M	I	[75]
1fyt	T	HA1.7	H	II	[76]
1j8h	T	HA1.7	H	II	[77]
1kj2	T/C	KB5-C20	M	I	[78]
1mi5	T	LC13	H	I	[79]
1mwa	T/C	2C	M	I	[80]
1nam	T/C	BM3.3	M	I	[81]
1oga	T/C	JM22	H	I	[82]
1qse	T	A6	H	I	[35]
1u3h	T	TCR172.10	M	II	[83]
2bnq	T/C	1G4	H	I	[37]
2bnr	T/C	1G4	H	I	[37]
2e7l	T/C	2C m6 [T7]	M	I	[84]
2esv	T/C	KK50.4	H	I	[85]
2f53	T/C	1G4 c49c50	H	I	[38]
2f54	T/C	1G4 AV-wt	H	I	[38]
2gj6	T/C	A6	H	I	[73]
2iam	T	E8	H	II	[86]
2ian	T	E8	H	II	[86]
2nx5	T/C	ELS4	H	I	[87]
2oi9	T/C	2C [T7-wt]	M	I	[84]
2ol3	T/C	BM3.3	M	I	[88]
2p5e	T/C	1G4 c58c61	H	I	[39]
2p5w	T/C	1G4 c58c62	H	I	[39]
2pxy	T	1934.4	M	II	[89]
2pye	T/C	1G4 c5c1	H	I	[39]
2vlk	T/C	JM22	H	I	[90]
2vlr	T/C	JM22	H	I	[90]
3c5z	T	B3K506	M	II	[91]
3c60	T	YAe62	M	II	[91]
3c6l	T	2 W20	M	II	[91]
3d39	T/C	A6	H	I	[92]
3d3v	T/C	A6	H	I	[92]
3dxa	T/C	DM1	H	I	[93]
3e2h	T/C	2C m67 [T7]	M	I	[65]
3e3q	T/C	2C m13 [T7]	M	I	[65]
3ffc	T/C	cf34	H	I	[94]
3gsn	T/C	RA14	H	I	[95]
3h9s	T/C	A6	H	I	[96]
3kpr	T/C	LC13	H	I	[97]
3kps	T/C	LC13	H	I	[97]
3kxf	T/C	SB27	H	I	[98]
3mbe	T	TCR 21.30	M	II	[99]
3mv8	T/C	TK3 Q55H	H	I	[100]
3pwp	T/C	A6	H	I	[101]
3qiu	T	226 TCR	M	II	[102]
3qiw	T	226 TCR	M	II	[102]

a) T: Structure only in dataset DS_T_. T/C: Structure in both datasets, DS_T_ and DS_C_

b) Species (S): *H* human, *M* mouse

c) Ligand type (L): MHC class I or II. See Additional file 4: Table S1 of the Supporting Information for details about the MHC alleles and the different peptides

d) References

We further derived three different data sets. First, to evaluate the performance of our method for the remodeling of the association angle of the TCR Vα and Vβ domains, we removed the pMHC ligand in each structure. This resulted in a set of 75 Vα/Vβ complexes, which we shall refer to as DS_T_ in the following. In addition, we created a second data set, in which only the first BU in the PDB file of the corresponding structure was included (48 structures, further referred to as DS_T_^*^). Then, to perform the remodeling of TCRpMHC complexes, we selected among the 75 BUs only the structures containing an MHC class I molecule. The resulting third data set, named as DS_C_, contains a total of 53 TCRpMHC complexes. We disregarded MHC class II molecules in the DS_C_ set to ensure a proper comparison between the samples. A third set could have been dedicated to MHC class II molecules. However, we sustained from remodeling also that set as the results obtained for the MHC class I complexes already showed that further optimization of the pipeline, beyond this publication, is necessary for accurate pMHC placement. In addition, the size of the MHC class II set (22 structures) would have been very small for a robust analysis. Further details about each data set are listed in Table S1 of the Supporting Information (Additional file 4: Table S1).

## Results and discussion

The structural prediction of immunologically relevant molecular assemblies has focused the interest of a wide range of methodological developments over the past decades, especially in the field of antibody-antigen interactions [41, 48, 49, 70]. Compared to the effort made so far in antibody modeling, the number of predicted TCRpMHC structures is still relatively small, as we discussed in the Background section. In the case of antibodies, it was recently shown by some of us, that statistical learning techniques can efficiently predict the V_H_/V_L_ association angles [49]. Such very appealing approaches are based on experimentally observed structural features and require a large amount of existing data. In the particular case of antibodies, over 2000 crystal structures are already available, thus allowing the application of such knowledge-based methodologies. Considering the relatively small amount of TCR structures referenced in the Protein Data Bank (i.e., about 200), such a road can unfortunately not be envisaged for the prediction of association angles in TCR complexes. As a consequence, we developed here a solely force-field based optimization strategy for TCR and TCRpMHC complexes modeling. Such a force-field based approach can potentially be applied to other similar systems, even if a sufficient amount of experimental data is not available for the training of a specific scoring function.

As we extensively described in the Background and in the Methods sections, this new algorithm, named as DynaDom, is derived from our previous comprehensive analysis of the Vα/Vβ TCR variable domain association angles [43]. The main conclusions that arose from this former work can be summarized as follows: i) TCR complexes can be classified into structural clusters, differing significantly in their Vα/Vβ inter-domain angles, ii) the angular differences between the structural clusters can be described by a simple rotation around a center of rotation (CoR_β_, see the Methods section and Fig. 1 for details), and iii) the CoR_β_ is characterized by two highly conserved glutamine residues, which contribute to the interaction between the TCR Vα and Vβ domains via a stabilizing hydrogen bond network.

For the remodeling of TCRpMHC complexes, the DynaDom method uses a unified cuboid description of the three different units of this complex (i.e., Vα, Vβ, pMHC). The optimization of the total system is performed by a rotation-based algorithm, which is based on our Center of Rotation concept (i.e., CoR_β_ and CoR_μ_, as described in the Methods section). In our previous analysis study, we observed that the Vα/Vβ association angle spectrum is much larger in unbound TCRs than in structures bound to the pMHC [43]. Pierce et al. further emphasized that for the prediction of TCRpMHC complexes from unbound units [33] the side chains of the CDR loops must adapt to their environment in order to allow for a proper interaction between the different units of the complex. Therefore, the inclusion of local side chain flexibility at the domain interface would most likely be a necessary extension for the prediction of TCRpMHC structures from unbound or homologous TCR and pMHC structures by homology modeling techniques. Our generalized, modular implementation ensures that the additional inclusion of local flexibility is straightforward. However, the adaptation of the algorithm would require additional extensive evaluation efforts, which would go beyond the scope of the present work and will be part of future investigations. Nevertheless, in the present work we already tested such a feature by the inclusion of local side chain flexibility for the two Q-Q residues at the CoR_β_, which we found to be crucial for the prediction success as we shall discuss in the following subsections.

The current version of our pipeline algorithm, results from an extensive series of evaluations intended to assess the effect of the different parameters. Hereafter, we present and discuss our main findings together with the actual evaluation of the method. We first discuss the optimization of the orientation of the glutamine residues (Q-flip correction) located at the interface between the two TCR variable domains (i.e., at the Center of Rotation CoR_β_ of Vβ with respect to Vα), based on the original experimental structures. Then, we analyze the effect of such a Q-flip correction together with the use of restraints on the remodeling of Vα/Vβ TCR and TCRpMHC complexes. Along this analysis, we compare the results obtained using either an energy or a structure based selection criterion (i.e., C_E_ or C_R_, respectively, as defined in the Methods section). This comparison intends to state if an atomistic force field energy based criterion could be used for future blind homology modeling of TCRpMHC complexes. We finally suggest further possible routes of improvement for our methodology, based on the analysis of the few cases in which the remodeling process did not lead to a satisfactory structure.

### Glutamine orientation correction

The interface between the Vα and Vβ domains of TCRs is characterized by the interaction of two highly conserved glutamine (Q) residues [43]. While this Q-Q interaction appears to be of critical importance, the flip state of these residues is often wrongly assigned in experimental crystal structures, due to the ambiguous character of the carboxamide group electron density [50–53]. In the context of this work, we analyzed the flip state of the Q residues among the crystal structures contained in our set of 75 Vα/Vβ complexes (i.e., data set DS_T_). Only 72.7% of the structures present a correct assignment of the Q-flip state. The details of this analysis are listed in Table S2 of the Supporting Information (Additional file 5: Table S2).

As discussed in the Background section, many modeling tools exist to correct the orientation of glutamine and asparagine residues in a given crystal structure. Among those, we tested Reduce [50, 54, 55] and Protoss [60, 61] on our DS_T_ set. The application of the Reduce and Protoss programs leads to an improvement of the glutamine flip state in our set, reaching 94.6 and 97.3% of correctly assigned Q-flip states, respectively. Analysis of the failed cases showed that they featured an interaction of the Q residues in an initial bifurcated orientation (i.e., associated in a perpendicular manner). Manual inspection showed that in these cases the perpendicular orientation allowed for optimal interactions with the rest of the domains and should therefore be the most stable in the functional receptor (i.e., not a further artifact of the carboxamide assignments in the experimental structures). As the Reduce and Protoss programs only allow parallel orientations, these cannot successfully predict such particular interactions. Because of this limitation and the below discussed observation that the Q-flip state can change upon the association of the Vα/Vβ domains, we decided to implement an independent Q-flip correction approach using our already implemented rigid body operators, such that it can directly be included into our pipeline. This represents a first probing of the modular character of our implementation, which we shall follow towards the future inclusion of local flexibility.

**Fig. 3:**
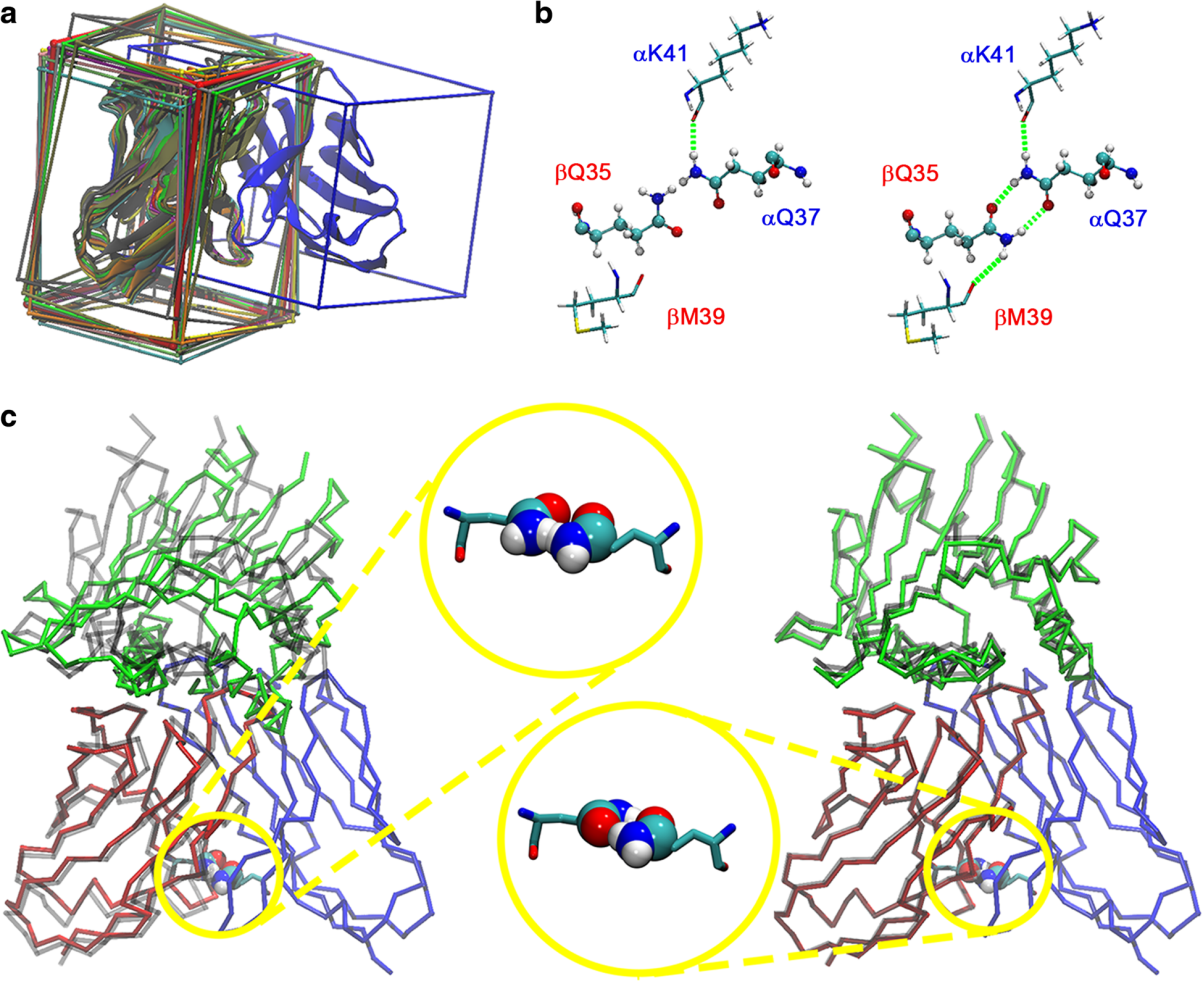
Remodeling of the 2f53 structure. **a** Superposition of the 11 Vβ starting orientations with respect to the Vα domain (represented in *blue* color). The average conformation of Vβ is shown in *red* color. **b** Hydrogen bonds of the conserved Q-Q interaction at the CoR_β_ position. Left: misassigned conformation in the experimental crystal structure. Right: proper orientation of the Q residues after application of the Q-flip correction. The picture shows that the interaction between the two residues has been improved as well as the interaction of the residues with their respective environment. **c** Modeling of the ternary TCRpMHC complex. The Vα, Vβ, and pMHC units are represented in *blue*, *red*, and *green* colors, respectively. The reference crystal structure is depicted in *gray* color. Left: initial assembly of the complex. Right: final model with an RMSD of 0.61 Å with respect to the crystal structure. Magnifications lenses: conformation of the conserved Q-Q interaction between the Vα and the Vβ domain

We evaluated the performance of our method for the Q-flip correction in the crystal structure of the DS_T_ set. We present here the most relevant findings of our analysis, while a more detailed discussion can be found as Supporting Information (Additional file 6: Text S3), together with the entirety of our observations per structure (Additional file 5: Table S2, Additional file 7: Table S3, and Additional file 8: Table S4). Using the DynaDom correction module, 100.0% of the structures could be assigned in the good Q-flip state. An example of a successful Q-flip correction is depicted in Fig. 3b for the 2f53 crystal structure. This higher performance obtained by our method with respect to the other programs comes from the optimization-based methodology that we implemented. While standard tools only consider two possible parallel conformations per residue (i.e., the original and the flipped state), DynaDom performs an explicit sampling of the carboxamide group using adjustable angular step sizes, followed by an energy minimization step during which the atomistic environment of the residue is taken into account. Such a protocol allows the system to escape local minima in order to find the most favorable conformations of the Q residues in their environment. For this reason, the DynaDom approach can also lead to a proper paring in the case of the two systems that present a bifurcated Q-Q interaction.

In a second step, we assessed the steps of our pipeline at which this correction should be performed. For this we applied our tool on the two TCR variable domains independently and compared the results with the same calculation performed in the Vα/Vβ complex environment (Additional files 7: Table S3 and Additional file 8: Tables S4). In this analysis, we observed different predicted Q-flip states if the corrections were applied on the single domains or on the final complexes. This observation emphasizes the impact of the environment of the glutamine residues on their respective conformation. For this reason, our standard pipeline algorithm performs the Q-flip state correction at two different steps. First, the glutamine residues are optimized accounting only for their respective domain environment (i.e., Vα or Vβ). This allows for a proper orientation of the glutamine residue prior to the complex assembly, thus eliminating potential errors originating from the experimental structures. It is noteworthy that this feature will be particularly relevant if homology modeled structures are built. Then, further optimizations are performed in the complete environment during the Vα/Vβ complex optimization to adapt the Q residues to the final relative orientation of the Vα and Vβ TCR domains.

### Modeling of the TCR variable domain complexes without pMHC

We tested our DynaDom methodology first for the remodeling of the Vα/Vβ association angles in the complete DS_T_ set. In particular, we performed a series of evaluations to assess the relevance of two criteria in our algorithm pipeline: the use of the Q-flip correction and the application of a distance restraint between Vα and Vβ. Furthermore, we analyzed the performance of our method according to two selection criteria. For a given remodeling experiment, DynaDom produces 11 models, which are ranked by their RMSD or energy score (see Additional file 9: Figure S1 Fig. and the description in the Methods section). The remodeling experiment is then counted as successful if the RMSD of the selected model with respect to the original crystal structure is lower than 2 Å.

Our results are summarized in Table 2 and the different evaluation settings that we considered are generally labeled as M_T_ plus a bit string, which encodes for the use or not of the Q-flip correction and the restraint (e.g., M_T_10 labels the remodeling of a TCR complex by applying the Q-flip correction but no distance restraint). The last M_T_^*^11 test corresponds to the M_T_11 settings performed for the DS_T_* data set, which contains only the first BU of each experimental structure.

**Table 2:** Prediction accuracy for the Vα/Vβ association angles modeled without pMHC

ES^a^	Variants^b^		C_R_^c^		C_E_^d^	
	Q	R	#	%	#	%
M_T_00	off	off	71	94.7	63	84.0
M_T_01	off	on	71	94.7	66	88.0
M_T_10	on	off	72	96.0	64	85.3
M_T_11	on	on	72	96.0	67	89.3
M_T_^*^11	on	on	47	97.9	43	89.6

^a^Evaluation setting label

^b^Variants: Q = glutamine carboxamide group orientation correction, R = rigid body position restraint

^c^Absolute and relative prediction rate according to the RMSD based criterion (i.e., C_R_) in data set DS_T_ (75 structures). In the particular case of M_T_^*^11, the prediction was performed on the DS_T_^*^ set (48 structures, without biological units). For each prediction run, the 11 models are ranked by RMSD and a success is counted if the selected structure has an RMSD value lower than 2 Å

^d^ Same as c) using the energy criterion to rank the 11 structures and select the best. The prediction is considered as successful if the selected structure has an RMSD value lower than 2 Å

Considering the C_R_ criterion, the remodeling procedure already reaches a very high positive prediction rate of 94.7%, even if no Q-flip corrections or restraints are used (M_T_00). This rate further increases to 96.0% if the Q-flip correction is switched on (M_T_10), while no change is observed if the distance restraint is used alone (94.7% in the M_T_01 case). As a consequence, the final prediction rate, with both parameters switched on, also reaches the remarkable rate of 96.0% (M_T_11). Only three outliers are observed, originating from the 3dxa and from the 1mwa crystal structures. The relatively low resolution of the 3dxa structure (i.e., 3.5 Å) can partially explain this failure. Furthermore, our modeling process only considers the Vα and Vβ domains of the TCR complex. It is possible that the two constant domains of the complex play an important role in these three outlier cases. Regarding the additional experiment performed on the DS_T_^*^ data set (M_T_^*^11), the results in Table 2 show that the differences in the achieved accuracies with respect to M_T_11 are only marginal. This confirms that the inclusion of the BUs does not bias the overall results and demonstrates the robustness of our algorithm with respect to small variations in the structures, thus highlighting the suitability of the approach in a future homology modeling pipeline.

In the perspective of a blind homology modeling experiment of TCR complexes, no reference crystal structure would be available and only an energy-based criterion could be considered for structure selection (i.e., C_E_). Based on such a C_E_ criterion, our remodeling attempt reaches a prediction rate of 84.0% even if no Q-flip corrections or restraints are applied (M_T_00). The prediction rate increases with both, the independent use of the Q-flip correction and the distance restraint to 85.3 and 88.0% for M_T_10 and M_T_01, respectively. If both parameters are used (M_T_11), the prediction reaches the remarkable rate of 89.3% and even 89.6% for the M_T_^*^11 data set. This last result is very promising for the further applications of the DynaDom method in a real structure prediction setting.

It appears that the use of the distance restraint has a stronger impact on the prediction rate obtained according to the C_E_ criterion than it has for the C_R_ criterion. This could be attributed to the observation that without distance restraint, the algorithm can yield structures in which the two TCR domains are placed in an unrealistic conformation, which nevertheless has a lower interaction energy (see Additional file 10: Figure S2). Such unphysical associations are far from the original crystal structure and are intrinsically discriminated by an RMSD based selection criterion.

**Fig. 4:**
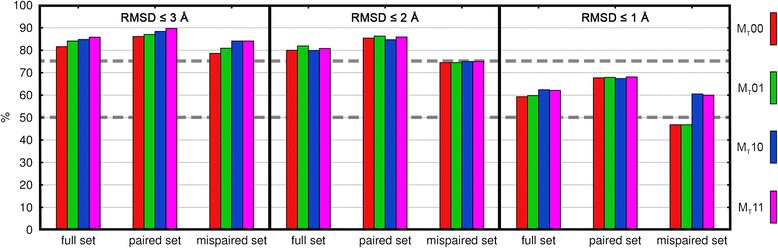
Percentage of structures among the 75*11 models with an RMSD value lower than 3, 2 and 1 Å. The total set of 75*11 structures is separated into structures for which the Q residues were originally paired or mispaired within their corresponding crystal structure. Each histogram box corresponds to a different setting of the modeling procedure, i.e. with only distance restraint (M_T_01), only Q-flip correction (M_T_10), both (M_T_11), or none of them (M_T_00). The percentage of structures with an RMSD value lower than 3, 2 and 1 Å are presented on the left, middle, and right plots, respectively. The right plot shows that for the structures presenting an originally wrong orientation of the Q residues, the Q-flip correction significantly improves the quality of the resulting model (i.e., M_T_10 and M_T_11)

Next to the analysis of the best conformations according to the C_E_ and C_R_ criteria we also analyzed the overall performance of the algorithm regarding the quality of all predicted conformations. In Fig. 4, we present the percentage of structures having an RMSD value lower than 1, 2 and 3 Å, depending on the algorithm settings (i.e., M_T_00, M_T_01, M_T_10, and M_T_11) among all 75*11 models produced by our DynaDom procedure. In this context we also further analyzed the impact of the Q-flip correction by classifying the resulting models into two groups, with respect to their original Q-flip state in the experimental structures as paired (51*11) and mispaired (20*11). Notice that 4*11 structures lack the presence of Q residues at CoR_β_ and were therefore not included in the respective analysis. The histograms (Fig. 4) show that an overall percentage of about 80% of the models feature an RMSD lower than 2 Å and thus fulfill our success criterion. This demonstrates the robustness of the presented algorithm and thus its relevance as one step in a comprehensive structure prediction pipeline. By further analyzing the influence of the Q-flip correction on the prediction rates, it can be observed that the overall prediction success is higher for structures in which the Q-Q orientation is already correct in the X-ray structure (paired structures). For these structures 85% of the models have an RMSD value lower than 2 Å, whereas the rate drops to 75% for the mispaired structures. This might be due to the relatively smaller size of the latter data set, as an investigation of a possible correlation between the crystal structure resolution and the quality of the final models did not yield any significant outcome.

Regarding the percentage of structures having an RMSD value lower than 2 and 3 Å for both sets, paired and mispaired, the results are practically independent on the defined settings and only a slight trend towards an improved performance can be observed if the Q-flip correction is applied. This low impact on the overall structures is most likely due to the large surface area of the total TCR domain interface and thus the high number of other interactions, which drive the overall optimization of the domains orientations. The use of the Q-flip correction has, however, a remarkable effect on the quality of the resulting structures once the cutoff is lowered to 1 Å. The percentage of models featuring such a low RMSD indeed increases from 47 to 60% for the mispaired structure set, if the correction is switched on. These observations reveal the importance of a correct orientation of the conserved Q residues at the Center of Rotation CoR_β_ for an accurate modeling of the TCR variable domain association and the need for their correction if they are wrongly assigned in the template structure.

Overall, this series of remodeling essays highlights the quality of our methodology. It also further emphasizes the applicability of a force field interaction energy-based criterion, which is very promising in the perspective of a homology modeling setting, as it shows that high-quality structures can be identified by this means.

### Modeling of the pMHC position with simultaneous TCR variable domain placement

Regarding the successful results obtained for the remodeling of TCR Vα/Vβ assemblies discussed in the previous subsection, we shall now assess the performance of the DynaDom method to remodel TCRpMHC complexes. Here, the calculations were performed on the smaller set of structures DS_C_, which contains a total of 53 biological units. Our results are listed in Table 3 and the labeling of the test settings follows the nomenclature introduced above (i.e., M_C_ label and a bit string for the use or not of Q-flip correction and distance restraint).

**Table 3:** Prediction accuracy for the combined prediction of the Vα/Vβ and TCR/pMHC association angles

ES^a^	Variants^b^		C_R_^c^		C_E_^d^	
	Q	R	#	%	#	%
M_C_00	off	off	39	73.6	37	69.8
M_C_01	off	on	39	73.6	38	71.7
M_C_10	on	off	39	73.6	38	71.7
M_C_11	on	on	39	73.6	38	71.7

^a^Evaluation setting label

^b^Variants: Q = glutamine carboxamide group orientation correction, R = rigid body position restraint

^c^Absolute and relative prediction rate according to the RMSD based criterion (i.e., C_R_) in data set DS_C_ (53 structures). For each prediction run, the 11 models are ranked by RMSD and a success is counted if the selected structure has an RMSD value lower than 2 Å

^d^Same as c) using the energy criterion to rank the 11 structures and select the best. The prediction is considered as successful if the selected structure has an RMSD value lower than 2 Å

An example of successfully predicted complex is depicted in Fig. 3c for the structure 2f53. In the figure, the Vα, Vβ, and MHC units are respectively colored in blue, red, and green. The two images represent the complex before and after optimization (on the left and on the right hand side of the figure, respectively). The magnifying glass shows that the Q-flip state is efficiently corrected and one can observe that the final model successfully fits the reference crystal structure (depicted with gray color in the picture).

Regarding the prediction rates according to the C_R_ and C_E_ criteria in Table 3, the percentages obtained for the remodeling of TCRpMHC complexes reach a less striking prediction rate, though still relatively high (i.e., 73.6 and 71.7% according to the C_R_ and C_E_ criteria, respectively). For the prediction based on the C_R_ criterion, the success rate appears to be independent on the use of Q-flip correction and distance restraints. A similar trend is observed with the C_E_ criterion. In this case, the use of one or both of the parameters only marginally increases the prediction rate. As for the modeling of TCR variable domains alone, the use of an energy based criterion yields very satisfactory results compared to a structure based one. This point also confirms the robustness and thus the suitability of our method for a blind homology modeling of TCRpMHC complexes.

### Detailed performance analysis for the TCRpMHC prediction

Regarding the overall, nearly equal, performances of the different settings in Table 3, it clearly appears that the drop of the prediction rate for the remodeling of TCRpMHC complexes with respect to the modeling of only the TCR variable domains is barely dependent on the use of Q-flip correction and distance restraints. The former parameters only affect the relative orientation of the Vα and Vβ domains. This observation indicates that the lower performance observed for the remodeling of TCRpMHC complexes might originate from an incorrect placement of the MHC molecule. We thus performed additional analyses to gain more insights into the shortcomings of the current approach and to identify potential routes for future improvement of our algorithm. To further confirm that the issues encountered in the remodeling of TCRpMHC complexes are solely due to the prediction of the pMHC positions with respect to the TCR, we analyzed the impact of the initial placement of the pMHC ligand on the remodeling of TCRpMHC complexes. In this context, we shall only consider the models obtained according to the RMSD based criterion (C_R_).

In the following series of test evaluations (T), we only consider the initial orientation of the TCR domains as found in their crystal structure (i.e., the remodeling procedure does not start from the 11 starting conformations, but only one). This was done to eliminate any potential biasing errors originating from the TCR domain modeling. Next to that, we used the settings of the final modeling pipeline: i.e.,Vβ optimization, Q-flip correction, and position restraints were systematically applied during these tests. As we described in the Methods section and depicted in Fig. 2, our modeling protocol includes a translation of the pMHC unit along a given axis to separate pMHC from the TCR, thus avoiding strong initial forces due to unphysical steric hindrance. This feature is one parameter that we shall analyze in the following tests (i.e., by switching it on or off). For each test setting, a rigid body optimization of the pMHC around its starting position was performed. Finally, for the first two test evaluations (T_1_ and T_2_), the MHC rigid body was initially placed in its crystal structure orientation, while for the last test (T_3_), this unit was oriented according to the general zero conformation discussed in the Method section (i.e., the orientation used in the standard pipeline). The results and details of each test evaluation are presented in Table 4.

**Table 4:** Prediction rates of the test evaluations

ES^a^	MHC initial	MHC	C_R_^d^	
	orientation^b^	translation^c^	#	%
T_1_	crystal	no	53	100.0
T_2_	crystal	yes	38	71.7
T_3_	general	yes	31	58.5

For each test the Q-flip correction as well as the use of distance restraint are systematically applied. The TCR Vβ domain is placed in its original crystal structure orientation and is optimized. The tests are performed for each of the 53 structures present in the DS_C_ data set and the MHC rigid body position is optimized in each case

^a^Evaluation setting label

^b^The initial orientation of the MHC unit is chosen either according to the original crystal structure or using the general zero orientation as for the standard version of our pipeline (see Methods Section for more details)

^c^Initial translation of the MHC unit to avoid steric hindrance, necessary if the MHC rigid body is not placed according to the crystal structure orientation (see Methods Section for more details)

^d^Absolute and relative prediction rate according to the RMSD based criterion (i.e., C_R_) in data set DS_C_ (53 structures). For each prediction run, a success is counted if the resulting model has an RMSD value lower than 2 Å with respect to the crystal structure

In the first test evaluation (T_1_) in which the pMHC units are oriented according to their respective crystal structure orientation and no initial translation is performed, the prediction rate reaches 100.0%. Although such a result could be expected as we start from the experimental conformations, it proves that our algorithm does not lead to any conformational artifacts. The additional application of the initial translation step for the pMHC ligand (T_2_) results in a drastic decrease of the prediction rate to 71.7%, slightly lower than the result obtained using our standard protocol (i.e., 73.6% in the M_C_11 case). For the final test (T_3_), in which the translated pMHC was placed according to our standard protocol, the prediction rate of our algorithm dramatically drops to 58.5%, which is significantly lower than for the final pipeline setting (73.6%).

These results show that the translation procedure and the preplacement of the pMHC ligand in a single general starting position constitute the accuracy limiting steps of our pipeline. In addition, we confirm here that the use of various starting positions for the Vβ domain clearly outperforms the case in which a single conformation is considered, even if the latter corresponds to the experimental crystal structure (i.e., 73.6% versus 58.6% for the M_C_11 and T_3_ cases, respectively). At first glance this is a surprising result. However, it clearly appears that the simultaneous optimization of both the TCR domains and the pMHC molecule as performed for the M_C_11, but not the T3 setting, is highly beneficial for the performance of the algorithm as it allows for an alternating adaption of the flexible units with respect to each other (Additional file 3: Movie S1). This leads to a smoother optimization path, thus lowering the probability for being trapped in a local minimum. Different starting positions further lower this probability as multiple paths are sampled.

Consequently, one straightforward way to improve our results should be to use multiple starting conformations for the pMHC ligand, in accordance with the 11 Vβ preplacement orientations. To evaluate this procedure, we chose one structure (PDB-ID 1oga) for which the modeling process failed in the last T_3_ test settings. For this structure, the three Euler angle components defining the pMHCs CoR_μ_-based rotational center were systematically varied by 5° and all 27 resulting starting poses were constructed. In accordance with the other test settings, the Vβ domain was here again placed in its crystal structure orientation. The results for the 27 resulting models are listed in the Supporting Information (Additional file 11: Table S5). The results improved considerably as this time five structures were obtained with an RMSD lower than 2 Å, thus satisfying our success criterion. Notably, these five models also show the lowest interaction energy among the 27 predicted structures. This last test clearly confirms the necessity of more advanced sampling protocol for the MHC molecule orientation in our modeling strategy to avoid the complex geometry to fall in an unfavorable local minimum. This is in agreement with the observations made by Pierce et al. [33] and demonstrates once again the importance of starting from multiple initial conformations. However, a straightforward combination of the 11 starting conformations of the TCR Vβ domain together with the 27 initial orientations of the pMHC unit would lead to a total of 297 structures to optimize per TCRpMHC complex, thus resulting in a dramatic increase of the computational cost.

Therefore, the presented algorithm provides excellent results and can readily be used for the optimization of the Vα/Vβ association angles. It also yields a fairly good prediction rate for the prediction of TCRpMHC complexes association. However, for the simultaneous optimization of both, the TCR domains and the placement of the pMHC in the latter case, further improvements and evaluations will be necessary prior to its practical use as one step in a real structure prediction pipeline. Considering the general, modular character of our implementation, also different approaches could be combined with the current method to tackle this issue. Among those, basin-hoping techniques [71] have proven to provide good results for the rigid body optimization of tryptophan zippers [72], and Monte Carlo-based rigid body sampling was recently applied by Pierce et al. for the placement of MHC like ligands alone [33, 34]. Despite the numerous tests that would be required for the combination of such techniques, this route represents a promising strategy for the future of our methodology.

## Conclusions

In this work we presented a new procedure, DynaDom, for the optimization of protein domain-domain orientations, which was designed for and evaluated on the special case of remodeling T-cell-receptor-peptide-MHC complexes. The approach is based on several rigid body optimization and restraining routines, and uses atomistic force field-based energy calculations. The individual optimization functions are combined in an application-specific pipeline. The method yields remarkable results for the remodeling of TCR Vα/Vβ association angles with prediction rates of 89–96% (RMSD < 2 Å) depending on the evaluation criterion.

The present study shows that it is possible to predict the TCR Vα/Vβ association angles on the basis of structural modeling only, without the need for a specially tailored experimental data dependent scoring function. It also demonstrates that the previously identified Center of Rotation concept [43] can be readily used for the structural prediction of the association angles.

Another striking result arising from this work is the observation that, by simply considering the best-energy conformation for each structure, high prediction rates of 89.3% for the Vα/Vβ association angles could be obtained. This is only marginally lower than the prediction rates obtained for the models with the smallest RMSD. This shows that ranking the modeled structures solely by their force field-based interaction energy allows the identification of high quality structures and demonstrates not only the robustness of the method, but also its suitability as part of a general structure prediction pipeline for TCRpMHC structures.

In a second step, we applied the concept to the simultaneous optimization of the TCR Vα/Vβ association angles and the pMHC positions on the TCR. However, due to efficiency considerations we used a simplified placement method for the pMHC, which resulted in lower prediction rates of 72–74%. This result is still in the predictive range, but not as high as for the TCR domain optimization. Additional preliminary investigations showed that the main reason lies indeed in the initial placement method of the pMHC ligand and that by simply using multiple initial conformations for the pMHC placement, already significant improvements in the placement accuracy are possible. However, a systematic optimization of the method for pMHC placement would require further significant evaluation studies, which would go beyond the scope of this manuscript and which will be the topic of future studies together with the application of DynaDom to the blind homology modeling of TCRpMHC complexes. In general, the presented approach is very well suited to serve as basis for the development of such a method for the prediction of atomistic models of TCRs or TCRpMHC complexes taking inter-domain angles into account. Due to the modular design of our program, a straightforward combination and concurrent optimization of multiple features is possible, as already demonstrated in this work by the concurrent optimization of the Vβ orientation, the pMHC orientation, and the adaption of the glutamine residues connecting the two TCR chains. Thus, the future implementation of partial or full flexibility of side chains or protein backbone regions, which then could be simultaneously optimized while the rigid body positions are adapted, should be straightforward. In addition, including other domains of the complex, such as the TCR constant domains would also be possible. This could help to study e.g. scissoring effects observed for the constant domains [73] or to investigate TCR signaling, which was elsewhere discussed to be induced by conformational changes in the constant domains [74].

Finally, it is worth noting that the DynaDom strategy is not limited to TCRpMHC assemblies. The combination of the different modules can indeed be easily modified to fit the requirement of other rigid body based predictions of a large variety of biomolecular assemblies.

## Additional files

Additional file 1: **Text S1.** Detailed discussion of important methodological aspects. (PDF 469 kb)

Additional file 2: **Text S2.** Detailed description of the operators. (PDF 134 kb)

Additional file 4: Table S1. Structural Dataset DS_T_ and the subset DS_C_. (PDF 99 kb)

Additional file 5: Table S2. Performance of the Q-Q interaction optimization. (PDF 92 kb)

Additional file 6: **Text S3.** Glutamine correction and adaption (detailed Results and Discussion). (PDF 99 kb)

Additional file 7: **Table S3.** Per residue flip states using Reduce, Protoss and DynaDom comparing single domains and TCR complexes. (PDF 145 kb)

Additional file 8 **Table S4:** Angular deviations with respect to the crystal structures after DynaDom glutamine refinement. (PDF 90 kb)

Additional file 9 **Figure S1:** Discrimination of the models. (PDF 1267 kb)

Additional file 10: **Figure S2:** Influence of the restraint operator. (PDF 625 kb)

Additional file 11: **Table S5:** pMHC optimization for the structure 1oga with different pMHC start conformations. (PDF 73 kb)

## Abbreviations

BFGS: Broyden-Fletcher-Goldfarb-Shanno
BU: Biological Unit; crystallographically independent molecule in the asymmetric unit
CDR: Complementary Determining Region
C_E,_ C_R_: ranking Criterion based on the Energy or the RMSD, respectively
CoR_β_: CoR_μ_, Center of Rotation in repect to the TCR Vβ and the pMHC, respectively
DS_C_: Data Set TCRpMHC Complex
DS_T_*: Reduced DS_T_ containing only one BU per structure
DS_T_: Data Set TCR
MHC: Major histocompatibility complex
M_T,_ M_C_: Modeling run for the TCR test set or the complex test set, respectively
PDB: Protein Data Bank
pMHC: Peptide presented in a Major Histocompatibility Complex molecule
Q: Glutamine
RMSD: Root Mean Square Deviation
SO: Sub-process Operator
T1, T2, and T3: Test evaluations with different conditions
TCR: T Cell Receptor
V_H_, V_L_: antibody Variable domains of the Heavy and the Light chain, respectively
Vα/Vβ: Variable domain of the TCR α- and the β-chain, respectively
