# Supplementary material for: DynaDom: structure-based prediction of T cell receptor inter-domain and T cell receptor-peptide-MHC (class I) association angles
Source: BMC Struct Biol. 2017 Feb 2;17:2. doi: 10.1186/s12900-016-0071-7 (PMC5289058; doi:10.1186/s12900-016-0071-7)
Supplement: Supplementary file 2 — Text S1. Detailed discussion of important methodological aspects. (PDF 469 kb) [file 12900_2016_71_MOESM1_ESM.pdf]

DynaDom: Structure-based prediction of TCR inter-domain and TCR-pMHC association angles - T. Hoffmann, A. Marion, and I. Antes
Additional File 1

Text S1: Detailed discussion of important methodological aspects

A. Brief description of the cuboid concept, the definition of the reference frames, and the Center of Rotations.

The theoretical framework of the present modeling pipeline follows the methodology introduced in our former analysis study of the Vα/Vβ association angles in TCR complexes [1]. In that former analysis, the structure with the PDB-ID 2bnu was used as an arbitrary reference to superimpose all TCR structures in the data set on the Vα domain and thus the 2bnu coordinate system is further used as reference. The work performed in ref. [1] can be briefly summarized as follows: First, the TCR Vα:Vβ structures were superimposed onto conserved residues of the Vα domain. Second, unified cuboids were placed around the two variable domains based on the backbone atoms of the conserved residues. Third, a pairwise Euler angle distance matrix was computed for all Vβ cuboids with respect to the position of the fixed Vα domain. Clustering of the matrix elements led to six major structural clusters. Fourth, a Center of Rotation was identified by embedding grids into the Vβ cuboids and determining the grid point with the lowest deviation. The position of this center of rotation, denoted as CoR_{β}, is found in the 2bnu coordinate at: 28.5, -37.6, 55.5 Å, and is stabilized by two interacting Q residues.

In the present work, as our pipeline also addresses the prediction of the orientation of the pMHC ligand relative to the TCR, we used the above described cuboid method to further analyze the pMHC angular orientation relative to the Vα domains of the TCRpMHC complexes. In analogy to the Vβ domain, we determined a Center of Rotation CoR_{μ} for the pMHC molecules, which is used for the rigid body optimization (Cartesian position in the 2bnu coordinate system: 31.2, -48.2, 34.5 Å). This CoR_{μ} is located at the center of the MHC peptide-binding groove close to the geometric center of the peptide backbone. However, no conserved residue (in analogy to the conserved Q residues for CoR_{β}) can be determined for CoR_{μ}, because the CoR_{μ} is slightly shifted away from the pMHC complex and lies in the middle of the binding groove.

In our set of TCRpMHC complexes, we further identified the structure with the lowest angular deviation from all other pMHC structures. That is the biological unit (BU) containing the chains R, S, T, and P of the structure with the PDB ID 3e3q. Thus, we use the 3e3q:RSTP orientation of the pMHC ligand in the 2bnu coordinate system as reference conformation for the placement of pMHC ligands in the modeling pipeline.

As described in the main text, the modeling pipeline makes use of a pre-placement of the pMHC ligand to avoid initial clashes with the TCR. For this purpose, we used a point cloud of the MHC β-sheet backbone atoms to define a β-sheet-based plane with respect to the reference MHC (3e3q). For the pre-placement of the pMHC, an axis perpendicular to this plane is used with the axis vector defined within the 2bnu coordinate system (Cartesian coordinates in the 2bnu frame: -12.0, 32.7, 93.8 Å), along which the pMHC entity is translated 13Å away from the TCR to avoid initial, unphysical clashes.

B. Detailed discussion of the choice of 11 starting conformations for the DynaDom pipeline

As we discussed in the main text, the DynaDom strategy uses multiple starting orientations of the Vβ domain with respect to Vα for each remodeling process attempt. For the present application, we defined 11 different orientations based on our former analysis of Vα/Vβ association angles. For a brief summary of the concepts used therein see above Text S1.A and for more details please refer to [1]. This study was performed on a large set of 85 structures. In this former work, the analysis of the pairwise Euler angles distance matrix resulted in the identification of six structural clusters. These six cluster are labeled as cl1 to cl6 in ref. [1]. The 11 orientations used in the present work were derived from these clusters as follows:

- We included the representative structure of each cluster (6 orientations, labeled as cl1 to cl6 in Additional file 9: Fig. S1)
- We computed the average over the six representative structures (1 orientation, labeled as Av in Additional file 9: Fig. S1)
- The two largest clusters (cl4 and cl6 in ref. [1]) were spitted into 2 sub-clusters each. The representative structure of each sub-cluster was then included here (4 orientations, labeled as cl4a, cl4b, cl6a, and cl6b in Additional file 9: Fig. S1).

We chose to use more than the six main cluster representatives as starting structures for the modeling, in order to span the available conformations space as comprehensively as possible right at the beginning and thus to increase the efficiency of the modeling pipeline.

Matrix A presents the pairwise Euler distance matrix between the 11 clusters defined above. That matrix shows that all clusters differ from each other in the association angle of the two TCR variable domains. It also proves the relevance of these orientations as starting structures, as they cover a significantly large range of possible association modes.

We further illustrate the relevance of using 11 starting structures by two individual cases of TCR variable domains association (re-modeling of 2p5e and 1kj2). In each case, the pairwise root mean square deviation matrix (backbone RMSD of the Vβ domain) of each structure with respect to the others (Matrices B, C, D, and E), is computed before and after application of the modeling pipeline. The diagonal of these matrices shows the RMSD of the corresponding model with respect to the target crystal structure. In addition, the binding energy of the models is given for the final structures.
From the matrices of 2p5e and 1kj2 before application of the pipeline (Matrices B and D) it can be observed that the 11 starting structures differ considerably from each other. Some are already close to the target crystal structure (e.g., cl1 of 2p5e) and some can deviate by more than 5 Å.
The results after application of the DynaDom pipeline are system dependent (Matrices C and E). For illustration purposes here we chose a very well performing system (2p5e) and one mediocre one (1kj2). In the case of 2p5e re-modeling, most of the models converge to very similar structures, which all resemble the target crystal structure very well. Therefore, in that particular case, 9 over 11 models bear a very low pairwise RMSD value. However, in the case of 1kj2, only four models reach our

success criterion of 2 Å with respect to the crystal structure. Interestingly, in both examples, a starting structure bearing a low RMSD with respect to the crystal structure will not necessarily yield the best final model. This can be due to the presence of atomic overlap or repulsive interactions in the starting structures. Such steric hindrance cannot be known *a priori* and a good way to tackle such issue is to multiply the number of starting conformations, thus expecting that another starting point will reach a better solution.

When analyzing the interaction energies of the final structures, we first observe that the model with the best energy belongs also to those resembling the target geometry. Matrix E presents "X" entries for the energy of some structures. Those cases correspond to a very highly positive binding energy. Such large numbers result from an overlap of the atoms between the two TCR variable domains. This phenomenon can occur during the placement of the cuboids, and sometimes remains during the optimization, the system being trapped in this unphysical conformation. Future developments of our methodology will tackle this issue by using a softcore potential during optimization [2].

These two examples show the importance of using multiple starting structures for the DynaDom strategy, and for similar modeling techniques in general. In the case of a blind modeling of TCR and TCRpMHC complexes, the association angle between the two TCR variable domains would be unknown, further emphasizing the need of a wide set of starting conformations.

As described above and in the main text, each of the 75 TCR structures that we remodeled in this work belongs to one of the 6 structural clusters identified in ref. [1], and used here to define the 11 starting orientations of the variable domains. Consequently, for each remodeling attempt, the correct conformation of the complex might already be present in this set of 11 starting structures. We further analyzed our results to assess whether our pipeline suffers from any bias regarding this matter. For each remodeling attempt, we systematically disregarded the models derived from the starting orientation that corresponds to the cluster to which the modeled structure belongs. For example, in the case of the remodeling of 1kj2 we disregarded the model derived from cl5, as this TCR complex belongs to that structural cluster. As shown in Matrix E, removing this model from the set of 11 final structures does not affect the results, as the second best ranked model (cl6b) still presents an RMSD value below 2Å with respect to the original crystal structure. By performing this analysis on the complete set of 75 TCR complexes, we reached prediction rates of 88.0% and 94.7% according to the energy-based and RMSD-based criteria, respectively. This represents only a slight drop compared to our standard protocol, which reaches 89.3% and 96%, as detailed in the main text. This last test confirms the relevance of our set of 11 starting conformations and further emphasizes the robustness of the DynaDom strategy.

Matrix A. Pairwise Euler angle distance matrix (see Text S1.A) between the 11 clusters (Av, cl1, cl2, cl3, cl4, cl4a, cl4b, cl5, cl6, cl6a, and cl6b denote the 11 starting orientations).

	Av	cl1	cl2	cl3	cl4	cl4a	cl4b	cl5	cl6	cl6a	cl6b
Av	0.00										
cl1	12.20	0.00									
cl2	5.56	8.85	0.00								
cl3	8.32	13.00	8.93	0.00							
cl4	6.14	14.20	8.54	14.40	0.00						
cl4a	11.20	16.30	12.40	19.30	5.22	0.00					
cl4b	4.19	13.90	7.47	12.50	2.09	7.30	0.00				
cl5	5.69	15.20	9.08	4.95	11.50	16.70	9.44	0.00			
cl6	5.88	17.50	10.80	9.08	9.94	15.00	7.99	4.16	0.00		
cl6a	8.43	20.30	13.20	11.10	11.70	16.50	9.88	6.26	2.76	0.00	
cl6b	3.64	14.80	8.66	7.49	8.75	13.90	6.73	3.25	2.76	5.51	0.00

Matrix B. Pairwise RMSD (Å) matrix of the 11 starting structures (Av, cl1, cl2, cl3, cl4, cl4a, cl4b, cl5, cl6, cl6a, and cl6b denote the starting orientations) used for the re-modeling of 2p5e (biological unit DE), i.e. BEFORE application of the modeling pipeline. The values in the diagonal (highlighted in green) are the RMSD of the corresponding model with respect to the target crystal structure**.**

	Av	cl1	cl2	cl3	cl4	cl4a	cl4b	cl5	cl6	cl6a	cl6b
Av	3.353										
cl1	3.002	0.940									
cl2	1.164	2.645	2.936								
cl3	2.378	3.691	2.572	4.144							
cl4	1.755	3.530	2.072	4.120	3.729						
cl4a	3.269	4.258	3.385	5.609	1.542	4.351					
cl4b	1.174	3.406	1.664	3.538	0.614	2.155	3.656				
cl5	1.650	3.894	2.247	1.149	3.334	4.863	2.725	4.316			
cl6	1.428	4.189	2.360	2.101	2.797	4.300	2.208	0.959	4.574		
cl6a	2.055	4.875	2.913	2.485	3.221	4.675	2.661	1.379	0.698	5.252	
cl6b	0.917	3.503	1.908	1.896	2.505	4.018	1.915	0.950	0.700	1.397	3.901

Matrix C. Pairwise RMSD (Å) matrix of the 11 final structures (Av, cl1, cl2, cl3, cl4, cl4a, cl4b, cl5, cl6, cl6a, and cl6b denote the starting orientations) obtained by the re-modeling of 2p5e (biological unit DE), i.e. AFTER application of the modeling pipeline. The values in the diagonal (highlighted in green) are the RMSD of the corresponding model with respect to the target crystal structure**.** The last column is the final interaction energy (kJ/mol) of the corresponding model. The model having the lowest interaction energy is shown in bold font.

	Av	cl1	cl2	cl3	cl4	**cl4a**	cl4b	cl5	cl6	cl6a	cl6b	Delta E final
Av	0.476											-478.46
cl1	0.023	0.481										-477.05
cl2	0.091	0.079	0.514									-477.15
cl3	0.038	0.027	0.093	0.458								-478.21
cl4	0.018	0.021	0.090	0.024	0.466							-478.29
**cl4a**	**0.017**	**0.014**	**0.873**	**0.022**	**0.007**	**0.470**						**-478.49**
cl4b	0.034	0.031	0.105	0.024	0.028	**0.026**	0.461					-478.26
cl5	0.038	0.026	0.089	0.014	0.022	**0.022**	0.027	0.464				-478.41
cl6	6.692	6.706	6.688	6.709	6.695	**6.699**	6.716	6.710	6.607			-445.31
cl6a	6.839	6.853	6.836	6.856	6.842	**6.846**	6.863	6.857	0.181	6.747		-444.89
cl6b	0.097	0.101	0.166	0.081	0.088	**0.091**	0.073	0.087	6.713	6.859	0.394	-478.01

Matrix D. Pairwise RMSD (Å) matrix of the 11 starting structures (Av, cl1, cl2, cl3, cl4, cl4a, cl4b, cl5, cl6, cl6a, and cl6b denote the starting orientations) used for the re-modeling of 1kj2 (biological unit DE), i.e. BEFORE application of the modeling pipeline. The values in the diagonal (highlighted in green) are the RMSD of the corresponding model with respect to the target crystal structure**.**

	Av	cl1	cl2	cl3	cl4	cl4a	cl4b	cl5	cl6	cl6a	cl6b
Av	3.525										
cl1	2.994	5.408									
cl2	1.172	2.584	3.766								
cl3	2.327	3.658	2.504	2.421							
cl4	1.714	3.488	2.062	4.028	4.967						
cl4a	3.196	4.176	3.334	5.485	1.510	6.415					
cl4b	1.146	3.377	1.672	3.459	0.602	2.111	4.405				
cl5	1.619	3.886	2.226	1.145	3.262	4.760	2.665	2.367			
cl6	1.429	4.200	2.378	2.095	2.748	4.218	2.172	0.957	2.812		
cl6a	2.058	4.882	2.941	2.489	3.180	4.599	2.634	1.385	0.695	2.770	
cl6b	0.906	3.516	1.909	1.877	2.446	3.929	1.867	0.934	0.697	1.391	3.022

Matrix E. Pairwise RMSD (Å) matrix of the 11 final structures (Av, cl1, cl2, cl3, cl4, cl4a, cl4b, cl5, cl6, cl6a, and cl6b denote the starting orientations) obtained by the re-modeling of 1kj2 (biological unit DE), i.e. AFTER application of the modeling pipeline. The values in the diagonal (highlighted in green) are the RMSD of the corresponding model with respect to the target crystal structure**.** The last column is the final binding energy (kJ/mol) of the corresponding model ("X" entries represent high unphysical binding energies). The model having the lowest interaction energy is shown in bold font.

	Av	cl1	cl2	cl3	cl4	cl4a	cl4b	**cl5**	cl6	cl6a	cl6b	Delta E final
Av	5.313											-1677.81
cl1	9.405	10.541										X
cl2	3.680	6.491	5.643									X
cl3	3.747	10.494	4.966	1.785								-1698.61
cl4	3.011	8.464	2.183	3.835	4.720							X
cl4a	4.176	7.952	2.784	5.834	2.394	6.673						X
cl4b	3.012	8.464	2.183	3.835	0.002	2.394	4.719					X
**cl5**	**3.940**	**10.531**	**5.056**	**0.204**	**3.937**	**5.940**	**3.936**	**1.590**				**-1700.82**
cl6	0.287	9.470	3.692	3.463	2.939	4.229	2.939	**3.657**	5.039			-1680.31
cl6a	0.287	10.533	5.073	0.250	3.958	5.962	3.957	**0.046**	3.699	1.545		-1699.14
cl6b	3.766	10.488	4.966	0.033	3.835	5.835	3.834	**0.179**	3.483	0.224	1.755	-1699.88

C. Detailed discussion of the energy minimization settings and convergence criteria

The modeling pipeline described in the main text involves several energy minimization steps, which we will briefly discuss here. Following the scheme presented in Figure 2 of the main text, the pipeline steps 2, 4, 5, and 6 use the BFGS minimizer to optimize a given selection of the system. Each BFGS call uses a convergence criterion set on the norm of the objective function gradient (here, 0.01 kJ/mol/nm) and a tolerance parameters (0.0001 kJ/mol) for the line search part of the algorithm. The pipeline steps 2, 4, and 5 are solely preparation steps. In these cases, the structure is not intended to reach a perfect minimum, but only a reasonable starting structure for the main part of the algorithm, step 6. For these three preparation steps, the energy minimization either reaches the convergence criteria or not, but systematically leads to a more energetically favorable intermediate geometry used in the next modeling step.

Step 6 of the modeling pipeline is the core step of the DynaDom method. At this stage, the energy minimization is of great importance and we will discuss in more details three relevant cases. As described in the main text and in Figure 2 of the paper, our prediction pipeline performs steps 5 and 6 twice. At the first instance, the orientation of the glutamine residues is optimized with respect to the initial orientation of the TCR variable domains in the 11 starting structures. Then at step 6, the concurrent optimization of all the units of the system is performed for 50 steps. After that, the optimization is stopped and step 5 is called in between, allowing to find a better orientation of the glutamine residues with respect to the current association of the variable domains. Finally, step 6 is continued until convergence is reached or for a maximum of 2950 steps.

In Figure A provided below, we show the energy as a function of the minimization steps for three examples. In each case, we plotted the energy for the first and second instance of the minimization procedure in step 6 in red and blue. The final energy is also depicted with a plain horizontal gray line. In addition, a focus on the step 50 is given as an inset. Notice that between step 50 and step 51, the pipeline step 5 is performed again to find a better orientation of the glutamine residues, thus expecting a lower energy.

At first glance, we observe that the total energy of the system systematically decreases along the minimization process. The inset of each plot shows that the energy drops significantly between step 50 and step 51, confirming that the intermediate search for a glutamine orientation is relevant and effective.

In most of the modeling performed in this work, the energy fulfills the convergence criteria after a few hundred steps. Such an example is represented by the modeling of 3c6l (biological unit AB) in the cl2 orientation. In the first panel of Figure A, one can observe a fast decrease of the energy during the first 50 steps, even reaching a small plateau. Then, between step 50 and 51, the energy drops due to a better orientation of the glutamine residues. Finally, the minimization continues for about 120 steps, meets the convergence criteria and stops.

However, it can occur in some rare cases that the algorithm does not converge. Here we distinguish two situations that can lead to such phenomenon. The first situation is depicted in the bottom left panel of Figure A, for the modeling of 3h9s (biological unit DE) in the cl1 orientation. The first part of the minimization is similar to that of 3c6l (above described), with a decrease of the energy during the first 50 steps and a drop after the optimization of the glutamine orientation. However, although the

energy reaches a stable plateau after a thousand steps, the convergence criteria are not fulfilled. Such situations occur if the system reaches a very narrow minimum of the potential energy surface, leading to instabilities in the evaluation of the gradient and thus to a failure of the convergence. Among the 75*11 pipeline runs performed for the remodeling of TCR complexes, this situation represents only 4% of the cases. Further analysis of these cases show that the energy oscillates in the plateau region around a constant value. The standard deviation of these oscillations among the last 100 minimization steps is higher than the convergence criterion but lower than 0.1 kJ/mol. Considering the large size of the molecular systems treated in this work and the wide contact area between the TCR variable domains, such accuracy is legitimately acceptable. Consequently, we considered such geometry minimizations as converge and accepted the resulting models.

The last situation corresponds to unphysical geometries presenting dramatic overlap between the atoms of the two variable domains. This is illustrated at the bottom right panel of Figure A for the optimization of 1kj2 (biological unit DE) in the cl2 orientation (see also the discussion in Text S1, and Matrix E). In that case, the minimization starts with an extreme excess of energy, due to a large number steric clashes in the system. While it can occur that the system escapes this highly unfavorable area of the potential energy surface, it can also be trapped due to large barriers separating those overlapping domains from physically realistic areas on the potential surface. This is the case in the present example. The energy decreases along the optimization and even reaches a stable plateau. However, the convergence cannot be met due to strong numerical instability in this unphysical conformation. As the final score of a model in the DynaDom strategy is the binding energy, such cases are intrinsically disregarded by the method, as they show a very unfavorable interaction energy ("X" entries in Matrix E of text S1.B).

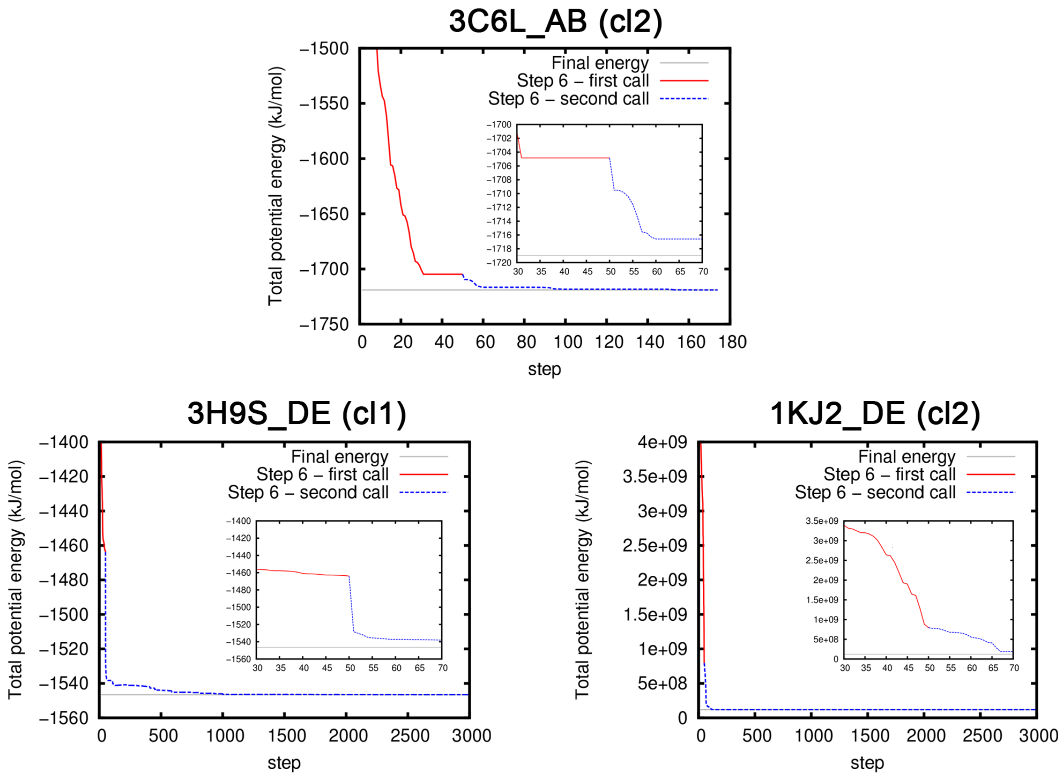

Figure A. Total potential energy as a function of the optimization steps for three different modeling cases (PDB-IDs 3C6L, 3H9S, and 1KJ2, with the biological units AB, DE, and DE, respectively). 3C6L_DE with the starting orientation cl2 illustrates a standard case in which the minimization converges rapidly. 3H9S_DE (orientation cl1) is a rare case in which the minimization process does not meet the convergence criteria, although the energy reaches a stable minimum. 1KJ2_DE (orientation cl2) is a structure that features an overlap between some atoms of the two TCR variable domains at the beginning of the procedure. In each case, the inset shows a focus around the minimization step 50, step at which the orientation of the glutamine residues is explicitly sampled and optimized.

References

1. Hoffmann T, Krackhardt AM, Antes I. Quantitative Analysis of the Association Angle between T-cell Receptor Valpha/Vbeta Domains Reveals Important Features for Epitope Recognition. PLoS computational biology. 2015;11(7):e1004244.

2. Antes I DynaDock: A new molecular dynamics-based algorithm for protein-peptide docking including receptor flexibility. Proteins. 2010 ;78(5):1084-1104.
