# Supplementary material for: DynaDom: structure-based prediction of T cell receptor inter-domain and T cell receptor-peptide-MHC (class I) association angles
Source: BMC Struct Biol. 2017 Feb 2;17:2. doi: 10.1186/s12900-016-0071-7 (PMC5289058; doi:10.1186/s12900-016-0071-7)
Supplement: Supplementary file 3 — Text S2. Detailed description of the operators. (PDF 134 kb) [file 12900_2016_71_MOESM2_ESM.pdf]

**DynaDom: Structure-based prediction of TCR inter-domain and TCR-pMHC association angles** - T. Hoffmann, A. Marion, and I. Antes
**Additional File 2**

## Text S2: Detailed description of the operators.

The implementation of the DynaDom approach is based on the definition of three basic operator functions, which are combined in the final prediction pipeline, which is discussed in the main text. Her we provide a detailed description of these functions.

**General objective function for the rigid body optimization operators:** All rigid body operators (RMZ, RAT, and RAR) operate on a selection $s_i$, which is transformed relatively to an additional part of the system defined as context $c_i$. For the evaluation of the objective function only non-bonded interactions between atoms of the $s_i$ and of the remaining context $c_i$ are taken into account. Internal non-bonded interactions within the selected rigid body are not computed, as they remain constant during rigid body operations. The contribution of the objective function $e_i$ is computed as follows:

$$e_{RXX,i} = \sum_{a \in s_i} \sum_{\substack{b \in c_i \\ b \notin s_i}} (E_{vdW}(a,b) + E_{Coul}(a,b)) \qquad (1)$$

In the context of the above described example: let $w_1$ and $w_2$ be atoms of the Vβ domain chain B, and $w_3$ and $w_4$ atoms of the Vα domain chain A. Then, $\{w_1, w_{2\}}\} \subseteq s_{TV\beta}$ is the selection and $\{w_1, w_2, w_3, w_4\} \subseteq c_{TV\beta}$ is the context, the intra-selection interactions ($w_1$, $w_2$) are ignored, whereas the inter-domain interactions ($w_1$, $w_3$), ($w_1$, $w_4$), ($w_2$, $w_3$), and ($w_2$, $w_4$) are taken into account.

**The rigid body rotation and translation operator (RMZ)** is based on the work of Mirzaei *et al.,* who recently introduced an algorithm focusing on the RBEM problem for molecular docking [2]. To minimize the interaction energy between two molecules, this algorithm rotates one molecule around a center (usually the center of mass), whereby this center is concurrently slightly translated to an optimum position. Their implementation makes use of the limited memory Broyden-Fletcher-Goldfarb-Shanno (L-BFGS) Newton-Raphson method [3] and of a transformation of the force field gradient based on the Rodrigues formula. The approach was shown to outperform other RBEM methods by an order of magnitude in time [2].
The operator takes the non-bonded interactions between the flexible rigid body and the defined context $c_i$ (without $s_i$) of the system into account, according to Equation 1. The operator simultaneously modifies three parameters for the rotation and three parameters for the translation of the rigid body. The gradient evaluation is discussed in detail in ref. [2]. We extended this method to allow for the simultaneous structural optimization of multiple rigid bodies. In the context of the TCRpMHC modeling, both the Vβ domain and the pMHC ligand are translated and rotated in one step.

**The rigid body axis operators RAT and RAR**: These operators define the separate translation along (RAT) or the rotation around (RAR) an axis according to the forces between the treated rigid body ($s_i$) and the remaining context $c_i$ without $s_i$. The objective function $e_i$ is computed according to Equation 1 of the main text.
To compute the gradient for the RAT operator, the non-bonded forces are projected on a predefined normalized axis ($\vec{A}$) as:

$$\overrightarrow{F_{RAT,i}} = \sum_{a \in s_i} \sum_{\substack{b \in c_i \\ b \notin s_i}} (\overrightarrow{F_{vdW}(a,b)} + \overrightarrow{F_{Coul}(a,b)}) \qquad (S1),$$

$$g_{RAT,i} = -\overrightarrow{F_{RAT,i}} \cdot \vec{A} \qquad (S2)$$

For the RAR operator the gradient is computed as the one-dimensional tangential force gradient:

$$F_t(s_i, c_i, \vec{A}, \vec{R}) = \sum_{a \in s_i} \sum_{\substack{b \in c_i \\ b \notin s_i}} \overrightarrow{T_{ab}^r} \cdot (1,0,0) \cdot \overrightarrow{D_a^r} \cdot (0,1,0) \qquad (S3)$$

$$g_{RAR,i} = -F_t(s_i, c_i, \vec{A}, \vec{R}) \quad \text{(S4)}$$

where $s_i$ denotes the atoms of the treated rigid body, and where $c_i$ is the remaining context to interact with. $\vec{R}$ is the coordinate vector of a predefined center of rotation and $\vec{A}$ defines the normalized rotation axis. $F_t$ is the one dimensional tangential force vector with respect to the rotation axis $\vec{A}$ for all atoms $a$ of the selection $s_i$ interacting with all atoms $b$ of the context $c_i$. The vector $\overrightarrow{T_{ab}^{r}}$ is the transformed projected force based on the objective function $e_i$, and $\overrightarrow{D_a^{r}}$ is the transformed distance vector of atom $a$ with respect with respect to the origin.

**The glutamine and asparagine carboxamide group rotation operator (AQR)** defines the rotation of a glutamine or asparagine carboxamide group around an axis defined in the direction of the bond between two adjacent side chain atoms (*i.e.* Cβ-Cγ and Cγ-Cδ for an asparagine and a glutamine residue, respectively). The selection $s_i$ ($s_i \subseteq c_i$) contains all atoms of the carboxamide group and the context is usually either the whole remaining protein or all atoms sharing the domain with the carboxamide group. The objective function contribution $e_i$ is computed as follows:

$$e_{AQR,i} = \sum_{a \in s_i} \sum_{\substack{b \in c_i \\ b \neq a}} (E_{vdW}(a,b) + E_{Coul}(a,b) + E_{bonded}(a,b)) \quad \text{(S5)}$$

The evaluation of the tangential force gradient for the AQR operator is based on Equations S3 and S4, but differs from the RAR evaluation in two aspects: First, for the RAR operator, interactions within the selection are not taken into account, whereas for the AQR these interactions are computed and second, also bonded interactions are considered.

**Rigid body position restraints (RPS and RPC):** To avoid unfavorable large translational moves, we implemented two types of restraint operators, which can be combined with the rigid body operators RAT and RMZ. The restraint operators allow for a certain tolerance of deviation and add distance dependent restraints to the translational gradients and to the objective function. The first type of restraint operator (RPS) restrains the coordinates of one single atom contained in the rigid body to be around a defined position. The second type (RPC) restrains the geometric center of a set of atoms contained in the rigid body to be around a defined position.
For both cases harmonic restraints are used and are computed as follows:

$$e_{rest} = \begin{cases} 0, & if\ d \leq t \\ \frac{k}{2} \cdot (d-t)^2 & \end{cases} \quad \text{(S6)}$$

where $t$ is the threshold, $d$ is the distance between the desired reference position of the rigid body ($\vec{R}$) and the actual position of the rigid body ($\vec{P}$) and $k$ an adjustable force constant (here, $k$ = 100.000 kJ $mol^{-1}$ $nm^{-2}$).
To restrain the position of the TCR Vβ domain, the RPS restraint operator is used. For this purpose, the reference position $\vec{R}$ is set to the Center of Rotation defined in ref. [1], the actual position $\vec{P}$ is defined as the coordinates of the $C_\beta$-atom of the $CoR_\beta$ Q residue, and the threshold is set to 0.75 nm.
To restrain the pMHC position, the RPC operator is used. As stated above, a conserved residue at the $CoR_\mu$ could not be found and the $CoR_\mu$ is located close to the center of the bound peptide. Due to the lack of a conserved residue, we define the actual position $\vec{P}$ of the rigid body pMHC as the geometric center between the C- and N-terminal $C_\alpha$-atoms of the bound peptide. The reference position $\vec{R}$ is set to the

$CoR_{\mu}$, and a threshold of 2.7 nm and 1.3 nm is used for the preplacement pipeline step and the main pipeline step, respectively (see below for the pipeline).

## References

1. Hoffmann T, Krackhardt AM, Antes I. Quantitative Analysis of the Association Angle between T-cell Receptor Valpha/Vbeta Domains Reveals Important Features for Epitope Recognition. PLoS computational biology. 2015;11(7):e1004244. doi: 10.1371/journal.pcbi.1004244. PubMed PMID: 26185983; PubMed Central PMCID: PMC4505886.

2. Mirzaei H, Beglov D, Paschalidis IC, Vajda S, Vakili P, Kozakov D. Rigid Body Energy Minimization on Manifolds for Molecular Docking. Journal of chemical theory and computation. 2012;8(11):4374-80. doi: 10.1021/ct300272j. PubMed PMID: 23382659; PubMed Central PMCID: PMC3561712.

3. Liu D, Nocedal J. On the limited memory BFGS method for large scale optimization. Mathematical Programming. 1989;45(1-3):503-28. doi: 10.1007/BF01589116.
