# Supplementary material for: DynaDom: structure-based prediction of T cell receptor inter-domain and T cell receptor-peptide-MHC (class I) association angles
Source: BMC Struct Biol. 2017 Feb 2;17:2. doi: 10.1186/s12900-016-0071-7 (PMC5289058; doi:10.1186/s12900-016-0071-7)
Supplement: Supplementary file 4 — Structural Dataset DST and the subset DSC. (PDF 99 kb) [file 12900_2016_71_MOESM4_ESM.pdf]

DynaDom: Structure-based prediction of TCR inter-domain and TCR-pMHC association angles - T. Hoffmann, A. Marion, and I. Antes

Additional File 4

Table S1: Structural Dataset DS_T and the subset DS_C (part 1).

BU[a]	D[b]	Name	S[c]	C[d]	Peptide	Ligand MHC I/II α	MHC II β
1AO7/DE	T/C	A6	H	I	LLFGYPVYV	HLA-A*0201	
1FO0/AB	T/C	BM3.3	M	I	INFDFNTI	H2-K1(b)	
1FYT/DE	T	HA1.7	H	II	PKYVKQNTLKLAT	HLA-DRA*0101	HLA-DRB1*0101
1J8H/DE	T	HA1.7	H	II	PKYVKQNTLKLAT	HLA-DRA*0101	HLA-DRB1*0401
1KJ2/AB	T/C	KB5-C20	M	I	KVITFIDL	H2-K1(b)	
1KJ2/DE	T/C	KB5-C20	M	I	KVITFIDL	H2-K1(b)	
1MI5/DE	T	LC13	H	I	FLRGRAYGL	HLA-B*0801	
1MWA/AB	T/C	2C	M	I	EQYKFYSV	H2-K1(bm3)	
1NAM/AB	T/C	BM3.3	M	I	RGYVYQGL	H2-K1(b)	
1OGA/DE	T/C	JM22	H	I	GILGFVFTL	HLA-A*0201	
1QSE/DE	T	A6	H	I	LLFGYPRYV	HLA-A*0201	
1U3H/AB	T	TCR172.10	M	II	SRGGASQYRPSQ	H2-Aa(u)	H2-Ab(u)
1U3H/EF	T	TCR172.10	M	II	SRGGASQYRPSQ	H2-Aa(u)	H2-Ab(u)
2BNQ/DE	T/C	1G4	H	I	SLLMWITQV	HLA-A*0201	
2BNR/DE	T/C	1G4	H	I	SLLMWITQC	HLA-A*0201	
2E7L/AD	T/C	2C m6 [T7]	M	I	QLSPFPFDL	H2-L(d)	
2E7L/BC	T/C	2C m6 [T7]	M	I	QLSPFPFDL	H2-L(d)	
2ESV/DE	T/C	KK50.4	H	I	VMAPRTLIL	HLA-E*0101	
2F53/DE	T/C	1G4 c49c50	H	I	SLLMWITQC	HLA-A*0201	
2F54/DE	T/C	1G4 AV-wt	H	I	SLLMWITQC	HLA-A*0201	
2F54/KL	T/C	1G4 AV-wt	H	I	SLLMWITQC	HLA-A*0201	
2GJ6/DE	T/C	A6	H	I	LLFGKPVYV	HLA-A*0201	
2IAM/CD	T	E8	H	II	GELIGILNAAKVPAD	HLA-DRA*0101	HLA-DRB1*0101
2IAN/DE	T	E8	H	II	GELIGTLNAAKVPA	HLA-DRA*0101	HLA-DRB1*0101
2IAN/IJ	T	E8	H	II	GELIGTLNAAKVPA	HLA-DRA*0101	HLA-DRB1*0101
2IAN/NO	T	E8	H	II	GELIGTLNAAKVPA	HLA-DRA*0101	HLA-DRB1*0101
2IAN/ST	T	E8	H	II	GELIGTLNAAKVPA	HLA-DRA*0101	HLA-DRB1*0101
2NX5/IJ	T/C	ELS4	H	I	EPLPQGQLTAY	HLA-B*3501	
2NX5/NP	T/C	ELS4	H	I	EPLPQGQLTAY	HLA-B*3501	
2NX5/TU	T/C	ELS4	H	I	EPLPQGQLTAY	HLA-B*3501	
2OI9/BC	T/C	2C [T7-wt]	M	I	QLSPFPFDL	H2-L(d)	
2OL3/AB	T/C	BM3.3	M	I	SQYYYNSL	H2-K1(bm8)	
2P5E/DE	T/C	1G4 c58c61	H	I	SLLMWITQC	HLA-A*0201	
2P5W/DE	T/C	1G4 c58c62	H	I	SLLMWITQC	HLA-A*0201	
2PXY/AB	T	1934.4	M	II	HSRGGASQYRPSQ	H2-Aa(u)	H2-Ab(u)
2PYE/DE	T/C	1G4 c5c1	H	I	SLLMWITQC	HLA-A*0201	
2VLK/DE	T/C	JM22	H	I	GILGFVFTL	HLA-A*0201	
2VLR/DE	T/C	JM22	H	I	GILGFVFTL	HLA-A*0201	
2VLR/IJ	T/C	JM22	H	I	GILGFVFTL	HLA-A*0201	
3C5Z/AB	T	B3K506	M	II	FEAQKAKANKAVD	H2-Aa(b)	H2-Ab(b)
3C5Z/EF	T	B3K506	M	II	FEAQKAKANKAVD	H2-Aa(b)	H2-Ab(b)
3C60/AB	T	YAe62	M	II	FEAQKAKANKAVD	H2-Aa(b)	H2-Ab(b)
3C60/EF	T	YAe62	M	II	FEAQKAKANKAVD	H2-Aa(b)	H2-Ab(b)
3C6L/AB	T	2W20	M	II	FEAQKAKANKAVD	H2-Aa(b)	H2-Ab(b)
3C6L/EF	T	2W20	M	II	FEAQKAKANKAVD	H2-Aa(b)	H2-Ab(b)
3D39/DE	T/C	A6	H	I	LLFGFPVYV	HLA-A*0201	
3D3V/DE	T/C	A6	H	I	LLFGFPVYV	HLA-A*0201	

a) Biological unit (BU) given as PDB ID and the two TCR chains as indicator.
b) T: only in DS_T; T/C: contained in both datasets, DS_T & DS_C.
c) Species: either human (H) or mouse (M).
d) MHC class presents in the original crystal structure.

Table S1: Structural Dataset DS_T and the subset DS_C (continued).

BU[a]	D[b]	Name	S[c]	C[d]	Peptide	Ligand MHC I/II α	MHC II β
3DXA/DE	T/C	DM1	H	I	EENLLDFVRF	HLA-B*4405	
3DXA/IJ	T/C	DM1	H	I	EENLLDFVRF	HLA-B*4405	
3DXA/NO	T/C	DM1	H	I	EENLLDFVRF	HLA-B*4405	
3E2H/BC	T/C	2C m67[T7]	M	I	QLSPFPFDL	H2-L(d)	
3E3Q/CF	T/C	2C m13[T7]	M	I	QLSPFPFDL	H2-L(d)	
3E3Q/de	T/C	2C m13[T7]	M	I	QLSPFPFDL	H2-L(d)	
3E3Q/DE	T/C	2C m13[T7]	M	I	QLSPFPFDL	H2-L(d)	
3E3Q/IJ	T/C	2C m13[T7]	M	I	QLSPFPFDL	H2-L(d)	
3E3Q/MN	T/C	2C m13[T7]	M	I	QLSPFPFDL	H2-L(d)	
3E3Q/RS	T/C	2C m13[T7]	M	I	QLSPFPFDL	H2-L(d)	
3E3Q/VW	T/C	2C m13[T7]	M	I	QLSPFPFDL	H2-L(d)	
3E3Q/Za	T/C	2C m13[T7]	M	I	QLSPFPFDL	H2-L(d)	
3FFC/DE	T/C	cf34	H	I	FLRGRAYGL	HLA-B*0801	
3FFC/IJ	T/C	cf34	H	I	FLRGRAYGL	HLA-B*0801	
3GSN/AB	T/C	RA14	H	I	NLVPMVATV	HLA-A*0201	
3H9S/DE	T/C	A6	H	I	MLWGYLQYV	HLA-A*0201	
3KPR/DE	T/C	LC13	H	I	EEYLKAWTF	HLA-B*4405	
3KPR/IJ	T/C	LC13	H	I	EEYLKAWTF	HLA-B*4405	
3KPS/DE	T/C	LC13	H	I	EEYLQAFTY	HLA-B*4405	
3KT/CF/D	T/C	SB27	H	I	LPEPLPQGQLTAY	HLA-B*3508	
3KXF/MO	T/C	SB27	H	I	LPEPLPQGQLTAY	HLA-B*3508	
3KXF/NP	T/C	SB27	H	I	LPEPLPQGQLTAY	HLA-B*3508	
3MBE/CD	T	TCR 21.30	M	II	GAMKRHGLDNY\ RGYSLGN	H2-Aa(d)	H2-Ab(NOD)
3MBE/GH	T	TCR 21.30	M	II	GAMKRHGLDNY\ RGYSLGN	H2-Aa(d)	H2-Ab(NOD)
3MV8/DE	T/C	TK3 Q55H	H	I	HPVGEADYFEY	HLA-B*3501	
3PWP/DE	T/C	A6	H	I	LGYGFVNYI	HLA-A*0201	
3QIU/CD	T	226 TCR	M	II	ADLIAYLKQATK	H2-Ea(k)	H2-Eb(k)
3QIW/CD	T	226 TCR	M	II	ADLIAYLEQATK	H2-Ea(k)	H2-Eb(k)

a) Biological unit (BU) given as PDB ID and the two TCR chains as indicator.
b) T: only in DS_T; T/C: contained in both datasets, DS_T & DS_C.
c) Species: either human (H) or mouse (M).
d) MHC class presents in the original crystal structure.
