# Supplementary material for: DynaDom: structure-based prediction of T cell receptor inter-domain and T cell receptor-peptide-MHC (class I) association angles
Source: BMC Struct Biol. 2017 Feb 2;17:2. doi: 10.1186/s12900-016-0071-7 (PMC5289058; doi:10.1186/s12900-016-0071-7)
Supplement: Supplementary file 5 — Performance of the Q-Q interaction optimization. (PDF 92 kb) [file 12900_2016_71_MOESM5_ESM.pdf]

DynaDom: Structure-based prediction of TCR inter-domain and TCR-pMHC association angles - T. Hoffmann, A. Marion, and I. Antes
Additional File 5

S2 Table: Performance of the Q-Q interaction optimization.

PDB	CH[a]		Pairing state[b]				PDB	CH[a]		Pairing state[b]			
ID	α	β	ES	RD	PT	DD	ID	α	β	ES	RD	PT	DD
1ao7	D	E	p	p	p	p	2vlr	I	J	p	p	p	p
1fo0	A	B	p	p	p	p	3c5z	A	B	p	p	p	p
1fyt	D	E	m	p	p	p	3c5z	E	F	p	p	p	p
1j8h	D	E	p	p	p	p	3c60	A	B	p	p	p	p
1kj2	A	B	p	p	p	p	3c60	E	F	p	p	p	p
1kj2	D	E	p	p	p	p	3c6l	A	B	m	p	p	p
1mi5	D	E	p	p	p	p	3c6l	E	F	m	p	p	p
1mwa	A	B	p	p	p	p	3d39	D	E	p	p	p	p
1nam	A	B	p	p	p	p	3d3v	D	E	p	m	p	p
1oga	D	E	p	p	p	p	3dxa	D	E	m	p	p	p
1qse	D	E	p	p	p	p	3dxa	I	J	m	p	p	p
1u3h	A	B	m	p	p	p	3dxa	N	O	m	p	p	p
1u3h	E	F	m	p	p	p	3e2h	B	C	p	p	p	p
2bnq	D	E	p	p	p	p	3e3q	C	F	p	p	p	p
2bnr	D	E	p	p	p	p	3e3q	d	e	p	p	p	p
2e7l	A	D	m	p	p	p	3e3q	D	E	p	p	p	p
2e7l	B	C	p	p	p	p	3e3q	I	J	p	p	p	p
2esv	D	E	n/a	n/a	n/a	n/a	3e3q	M	N	p	p	p	p
2f53	D	E	m	p	p	p	3e3q	R	S	p	p	p	p
2f54	D	E	m	p	p	p	3e3q	V	W	p	p	p	p
2f54	K	L	m	p	p	p	3e3q	Z	a	p	p	p	p
2gj6	D	E	p	p	p	p	3ffc	D	E	p	p	p	p
2iam	C	D	p	p	p	p	3ffc	I	J	p	p	p	p
2ian	D	E	p	p	p	p	3gsn	A	B	n/a	n/a	n/a	n/a
2ian	I	J	p	p	p	p	3h9s	D	E	p	p	p	p
2ian	N	O	p	p	p	p	3kpr	D	E	p	p	p	p
2ian	S	T	p	p	p	p	3kpr	I	J	p	p	p	p
2nx5	I	J	p	p	p	p	3kps	D	E	p	p	p	p
2nx5	N	P	p	p	p	p	3kxf	D	E	p	p	p	p
2nx5	T	U	p	p	p	p	3kxf	M	O	p	p	p	p
2oi9	B	C	p	p	p	p	3kxf	N	P	m	p	p	p
2ol3	A	B	p	p	p	p	3mbe	C	D	x	x	x	p
2p5e	D	E	m	p	p	p	3mbe	G	H	x	x	x	p
2p5w	D	E	m	p	p	p	3mv8	D	E	m	p	p	p
2pxy	A	B	m	m	p	p	3pwp	D	E	p	p	p	p
2pye	D	E	m	p	p	p	3qiu	C	D	n/a	n/a	n/a	n/a
2vlk	D	E	p	p	p	p	3qiw	C	D	n/a	n/a	n/a	n/a
2vlr	D	E	p	p	p	p							

a) TCR chain ID contained in the same biological unit.
b) Pairing states in different models (ES: experimental crystal structure; RD/PT: crystal structure after application of the REDUCE/Protoss program. DD: final model after full application of the DynaDom approach on the crystal structure): p=paired, m=mispaired, x=crossing conformation, n/a=not applicable due to missing CoR_{β} glutamine in one of the chains.
