# Supplementary material for: DynaDom: structure-based prediction of T cell receptor inter-domain and T cell receptor-peptide-MHC (class I) association angles
Source: BMC Struct Biol. 2017 Feb 2;17:2. doi: 10.1186/s12900-016-0071-7 (PMC5289058; doi:10.1186/s12900-016-0071-7)
Supplement: Supplementary file 6 — Text S3. Glutamine correction and adaption (detailed Results and Discussion). (PDF 99 kb) [file 12900_2016_71_MOESM6_ESM.pdf]

Text S3: Glutamine correction and adaption (detailed Results and Discussion).
One of the difficulties encountered in X-ray crystallography is to distinguish between neighboring oxygen and nitrogen atoms in electron density maps, since these two elements share a similar electron density. As a consequence, in many structures, the carboxamide atoms of glutamine and asparagine cannot be assigned to the correct coordinates with a high certainty and might by flipped by 180°. We investigated the conserved Q-Q interactions at the CoR_{β} position in the crystal structures of the dataset DS_T visually (Fig. 2B of the main text), with the MolProbity Reduce tool, and with the Protoss program. As listed in Table S2 (Additional file 5), we found several structures in which the glutamine residues were placed in a way that atoms of the same type (and thus of the same charge) were opposed to each other (1fyt/DE, 1u3h/AB and EF, 2e7l/AD, 2f53/DE, 2f54/DE and KL, 2p5e/DE, 2p5w/DE, 2pxy/AB, 2pye/DE, 3c6l/AB and EF, 3dxa/DE, IJ, and NO, 3kxf/NP, and 3mv8/DE). These presumed misplacements lead to unphysical repulsions between the two TCR chains, whereas in reality the glutamine residues form strong attractive electrostatic interactions induced by the interaction of the positively charged nitrogen and the negatively charged oxygen of the opposing residue. Furthermore, we found structural examples (1ao7/DE, 1fo0/DE, 1kj2/AB, 1mwa/AB, 1nam/AB, 1qse/DE, 2gj6/DE, 3kxf/DE) in which the glutamine residues indeed optimally interact with each other, but were both presumably flipped, as proposed by the Reduce tool and the Protoss tool (Additional files 7 and 8: Tables S3 and S4, columns CF). (Notably, the two different algorithms provided contradictory results for the structures 1nam, 2ol3, 2pxy, 3d3v, 3dxa, 3kxf, and 3mbe.) In these cases a rotation by 180° of both of the residues endgroups leads to a more favored interaction of these residues with the remaining protein. In most of these cases, the orientation of the Qα is determined by a hydrogen bond interaction between the amino terminal group of the glutamine and a backbone oxygen (Fig. 2C).
The application of Reduce[1-3] and Protoss[4,5] to the separated Vα and Vβ domains and to the Vα:Vβ complex showed that some TCRs adapt to different flip-states of the conserved CoR_{β} glutamine residues in the single chain state and in the complex state (see Additional Files 7 and 8: Tables S3 and S4, *e.g.* 1nam/AB, 1oga/DE, or 2gj6/DE). This result could be expected, as the glutamine residues at the CoR_{β} are surface residues in the individual chains and thus conformationally more flexible. Therefore a single preprocessing step involving only the individual chains is not suitable for an optimal placement of the conserved glutamine residues in the Vα:Vβ complex structure.
In order to address this issue, we developed a special procedure to correct the glutamine flip states concurrently before and during our rigid body optimization process. In this approach, both glutamine residues are first optimized only accounting for intra-domain interactions. Afterwards, the optimization is repeated by taking the inter-domain interactions into account as well. During the rigid body optimization both glutamine residues are free to rotate. However, a full rotation can be hampered by sterical clashes. To overcome this problem, we introduced a fast sampling step into our pipeline. For the correction of individual glutamine residues, in the sampling only the flipped and the unflipped states were taken into account. The sampling of both residues together was performed with smaller angular steps of 18°. After the sampling a short energy minimization rotating the amide groups was carried out.
In an intermediate pipeline step after 50 iterations of the main rigid body optimization, both glutamine residues were sampled and refined again. This readjustment of the two residues is used, since some initial rigid body orientations

prevent the formation of the bifurcation. In many cases a correct placement of the glutamine residues is only possible after some cycles of the rigid body optimization.
To validate our glutamine correction approach, we investigated the rotational states of the CoR_{β} glutamine residues before the rigid body optimization took place and compared the conformations of these residues with their counterpart found in the crystal structures (Additional file 5: Table S2). In all cases that show the assumed mispairing, at least one glutamine residue was flipped to allow the bifurcated Q-Q interaction. For all other structures in the DS_{T} dataset without an obvious mispairing, the Q-Q interaction was preserved. Our method performs similarly compared to the two methods used as a reference, namely Reduce and Protoss (Additional file 5: Table S2): With Protoss in all cases a pairing of the Q residues was reached. In contrast, the Reduce method did not correct the structure 2pxy/AB and introduced a mispairing in the structure 3d3v/DE. As a quantitative measure, we provide the pairing rates based on the number of structures containing two glutamine residues at the CoR_{β}: original crystal: 72.7%; Reduce: 94.6%; Protoss: 97.3%; DynaDom: 100%. Remarkably, our method was also able to transform the two orthogonal-like conformations into paired conformations.
Taking the correction of the bifurcated Q-Q interaction into account generally increases the prediction rate for the modeling of the TCR inter-domain angles as well as for the application on the TCRpMHC complex.

References

1. Chen VB, Arendall WB, 3rd, Headd JJ, Keedy DA, Immormino RM, Kapral GJ, et al. MolProbity: all-atom structure validation for macromolecular crystallography. Acta crystallographica Section D, Biological crystallography. 2010;66(Pt 1):12-21. doi: 10.1107/S0907444909042073. PubMed PMID: 20057044; PubMed Central PMCID: PMC2803126.
2. Davis IW, Leaver-Fay A, Chen VB, Block JN, Kapral GJ, Wang X, et al. MolProbity: all-atom contacts and structure validation for proteins and nucleic acids. Nucleic Acids Res. 2007;35(Web Server issue):W375-83. doi: 10.1093/nar/gkm216. PubMed PMID: 17452350; PubMed Central PMCID: PMC1933162.
3. Word JM, Lovell SC, Richardson JS, Richardson DC. Asparagine and glutamine: using hydrogen atom contacts in the choice of side-chain amide orientation. J Mol Biol. 1999;285(4):1735-47. doi: 10.1006/jmbi.1998.2401. PubMed PMID: 9917408.
4. Lippert T, Rarey M. Fast automated placement of polar hydrogen atoms in protein-ligand complexes. Journal of cheminformatics. 2009;1(1):13. doi: 10.1186/1758-2946-1-13. PubMed PMID: 20298519; PubMed Central PMCID: PMC3225823.
5. Bietz S, Urbaczek S, Schulz B, Rarey M. Protoss: a holistic approach to predict tautomers and protonation states in protein-ligand complexes. Journal of cheminformatics. 2014;6:12. doi: 10.1186/1758-2946-6-12. PubMed PMID: 24694216; PubMed Central PMCID: PMC4019353.
