# Supplementary material for: DynaDom: structure-based prediction of T cell receptor inter-domain and T cell receptor-peptide-MHC (class I) association angles
Source: BMC Struct Biol. 2017 Feb 2;17:2. doi: 10.1186/s12900-016-0071-7 (PMC5289058; doi:10.1186/s12900-016-0071-7)
Supplement: Supplementary file 7 — Table S3. Per residue flip states using Reduce, Protoss and DynaDom comparing single domains and TCR complexes. (PDF 145 kb) [file 12900_2016_71_MOESM7_ESM.pdf]

Table S3: Per residue flip states using Reduce, Protoss and DynaDom comparing single domains and TCR complexes (part 1).

PDB ID	CH[a] α	CH[a] β	RI[b] α	RI[b] β	Reduce *SF*[c] *α*	Reduce *SF*[c] *β*	Reduce CF[d] α	Reduce CF[d] β	Protoss *SF*[c] *α*	Protoss *SF*[c] *β*	Protoss CF[d] α	Protoss CF[d] β	DynaDom[e] *SF*[c] *α*	DynaDom[e] *SF*[c] *β*	DynaDom[e] SR[f] α	DynaDom[e] SR[f] β	DynaDom[e] *CR*[g] *α*	DynaDom[e] *CR*[g] *β*
1ao7	D	E	37	37	*K*	*F*	F	F	*F*	*F*	F	F	*F*	*F*	F	F	*F*	*F*
1fo0	A	B	37	37	*K*	*K*	F	F	*K*	*F*	F	F	*F*	*F*	F	F	*F*	*F*
1fyt	D	E	37	37	*F*	*K*	F	K	*F*	*K*	F	K	*K*	*K*	K	K	*F*	*K*
1j8h	D	E	37	37	*K*	*K*	K	K	*K*	*K*	K	K	*F*	*K*	F	K	*K*	*K*
1kj2	A	B	37	37	*F*	*F*	F	F	*F*	*F*	F	F	*F*	*F*	F	F	*F*	*F*
1kj2	D	E	37	37	*F*	*F*	F	F	*F*	*F*	F	F	*F*	*F*	F	F	*F*	*F*
1mi5	D	E	37	37	*K*	*K*	K	K	*K*	*K*	K	K	*F*	*K*	F	K	*K*	*K*
1mwa	A	B	37	37	*F*	*K*	F	F	*F*	*F*	F	F	*F*	*F*	F	F	*F*	*F*
1nam	A	B	37	37	*K*	*F*	K	K	*K*	*F*	F	F	*F*	*F*	F	F	*F*	*F*
1oga	D	E	38	39	*K*	*K*	K	K	*K*	*K*	K	K	*K*	*K*	K	K	*K*	*K*
1qse	D	E	37	37	*F*	*F*	F	F	*F*	*F*	F	F	*F*	*F*	F	F	*F*	*F*
1u3h	A	B	37	37	*K*	*F*	K	F	*K*	*F*	K	F	*K*	*F*	K	F	*K*	*F*
1u3h	E	F	37	37	*K*	*F*	K	F	*K*	*F*	K	F	*K*	*F*	K	F	*K*	*F*
2bnq	D	E	38	36	*F*	*F*	F	F	*F*	*F*	F	F	*F*	*F*	F	F	*F*	*F*
2bnr	D	E	38	36	*F*	*F*	F	F	*F*	*F*	F	F	*F*	*F*	F	F	*F*	*F*
2e7l	A	D	37	37	*K*	*F*	K	F	*K*	*F*	K	F	*K*	*F*	K	F	*K*	*F*
2e7l	B	C	37	37	*K*	*K*	K	K	*K*	*K*	K	K	*K*	*K*	K	K	*K*	*K*
2esv	D	E	36	n/a	*K*	*n/a*	K	n/a	*K*	*n/a*	K	n/a	*K*	*n/a*	K	n/a	*K*	*n/a*
2f53	D	E	37	35	*K*	*F*	K	F	*K*	*F*	K	F	*K*	*F*	K	F	*K*	*F*
2f54	D	E	37	35	*K*	*F*	K	F	*K*	*F*	K	F	*K*	*F*	K	F	*K*	*F*
2f54	K	L	37	35	*K*	*K*	K	F	*K*	*K*	K	F	*K*	*F*	K	F	*F*	*K*
2gj6	D	E	37	37	*F*	*K*	F	F	*F*	*K*	F	F	*F*	*F*	F	F	*F*	*F*
2iam	C	D	36	37	*K*	*K*	K	K	*K*	*K*	K	K	*K*	*K*	K	K	*K*	*K*
2ian	D	E	36	37	*K*	*K*	K	K	*K*	*K*	K	K	*K*	*K*	K	K	*K*	*K*
2ian	I	J	36	37	*K*	*K*	K	K	*K*	*K*	K	K	*K*	*K*	K	K	*K*	*K*
2ian	N	O	36	37	*K*	*K*	K	K	*K*	*K*	K	K	*K*	*K*	K	K	*K*	*K*
2ian	S	T	36	37	*K*	*K*	K	K	*K*	*K*	K	K	*K*	*K*	K	K	*K*	*K*
2nx5	I	J	37	37	*K*	*K*	K	K	*K*	*K*	K	K	*K*	*K*	K	K	*K*	*K*
2nx5	N	P	37	37	*K*	*K*	K	K	*K*	*K*	K	K	*K*	*K*	K	K	*K*	*K*
2nx5	T	U	37	37	*K*	*K*	K	K	*K*	*K*	K	K	*K*	*K*	K	K	*K*	*K*
2oi9	B	C	37	37	*K*	*K*	K	K	*K*	*K*	K	K	*K*	*K*	K	K	*K*	*K*
2ol3	A	B	37	37	*K*	*K*	F	F	*K*	*K*	K	K	*K*	*K*	K	K	*K*	*K*
2p5e	D	E	38	35	*K*	*F*	K	F	*K*	*F*	K	F	*K*	*F*	K	F	*K*	*F*
2p5w	D	E	38	35	*K*	*F*	K	F	*K*	*F*	K	F	*K*	*F*	K	F	*K*	*F*
2pxy	A	B	37	37	*F*	*K*	F	F	*F*	*K*	F	K	*F*	*K*	F	K	*F*	*K*
2pye	D	E	38	35	*K*	*F*	K	F	*K*	*F*	K	F	*K*	*F*	K	F	*K*	*F*
2vlk	D	E	38	39	*K*	*K*	K	K	*K*	*K*	K	K	*K*	*K*	K	K	*K*	*K*
2vlr	D	E	38	39	*K*	*K*	K	K	*K*	*K*	K	K	*K*	*K*	K	K	*K*	*K*
2vlr	I	J	38	39	*K*	*K*	K	K	*K*	*K*	K	K	*K*	*K*	K	K	*K*	*K*
3c5z	A	B	37	35	*K*	*K*	K	K	*K*	*K*	K	K	*K*	*K*	K	K	*K*	*K*
3c5z	E	F	37	35	*K*	*K*	K	K	*K*	*K*	K	K	*K*	*K*	K	K	*K*	*K*

a) TCR chain pairs indicating the biological unit.
b) Residue IDs of the conserved glutamine residues at the center of rotation.
c) Flip state, after separate application of the tool to individual chains.
d) Flip state, after application of the tool to the complex.
e) Flip state before rigid body optimization.
f) Flip state, after separate application of the refinement to individual chains (Threshold 90°).
g) Flip state, after application of refinement tool to the complex (Threshold 90°).
Angular values of the refinements are listed in Additional file 8: Table S4. K=keep; F=flip.

DynaDom: Structure-based prediction of TCR inter-domain and TCR-pMHC association angles - T. Hoffmann, A. Marion, and I. Antes
Additional File 7

Table S3: Per residue flip states using Reduce, Protoss and DynaDom comparing single domains and TCR complexes (continued).

PDB ID	CH[a]		RI[b]		Reduce				Protoss				DynaDom[e]					
					SF[c]		CF[d]		*SF*[c]		CF[d]		*SF*[c]		SR[f]		*CR*[g]	
	α	β	α	β	*α*	*β*	α	β	*α*	*β*	α	β	*α*	*β*	α	β	*α*	*β*
3c60	A	B	37	35	*K*	*K*	K	K	*K*	*K*	K	K	*K*	*K*	K	K	*F*	*K*
3c60	E	F	37	35	*K*	*K*	K	K	*K*	*K*	K	K	*K*	*K*	K	K	*F*	*K*
3c6l	A	B	37	35	*K*	*F*	K	F	*K*	*F*	K	F	*K*	*F*	K	F	*K*	*F*
3c6l	E	F	37	35	*K*	*F*	K	F	*K*	*F*	K	F	*K*	*F*	K	F	*K*	*F*
3d39	D	E	37	37	*F*	*F*	F	F	*F*	*F*	F	F	*F*	*F*	F	F	*F*	*F*
3d3v	D	E	37	37	*F*	*F*	F	K	*F*	*F*	F	F	*F*	*F*	F	F	*F*	*F*
3dxa	D	E	44	44	*F*	*K*	F	K	*F*	*K*	K	F	*F*	*K*	F	K	*F*	*K*
3dxa	I	J	44	44	*F*	*K*	F	K	*F*	*K*	K	F	*F*	*K*	F	K	*F*	*K*
3dxa	N	O	44	44	*F*	*K*	F	K	*F*	*K*	F	K	*F*	*K*	F	K	*F*	*K*
3e2h	B	C	37	37	*K*	*K*	K	K	*K*	*K*	K	K	*K*	*K*	K	K	*K*	*K*
3e3q	C	F	37	37	*K*	*K*	K	K	*K*	*K*	K	K	*K*	*K*	K	K	*K*	*K*
3e3q	d	e	37	37	*K*	*K*	K	K	*K*	*K*	K	K	*K*	*K*	K	K	*K*	*K*
3e3q	D	E	37	37	*K*	*K*	K	K	*K*	*K*	K	K	*K*	*K*	K	K	*K*	*K*
3e3q	I	J	37	37	*K*	*K*	K	K	*K*	*K*	K	K	*K*	*K*	K	K	*K*	*K*
3e3q	M	N	37	37	*K*	*K*	K	K	*K*	*K*	K	K	*K*	*K*	K	K	*K*	*K*
3e3q	R	S	37	37	*K*	*K*	K	K	*K*	*K*	K	K	*K*	*K*	K	K	*K*	*K*
3e3q	V	W	37	37	*K*	*K*	K	K	*K*	*K*	K	K	*K*	*K*	K	K	*K*	*K*
3e3q	Z	a	37	37	*K*	*K*	K	K	*K*	*K*	K	K	*K*	*K*	K	K	*K*	*K*
3ffc	D	E	44	44	*K*	*K*	K	K	*K*	*K*	K	K	*K*	*K*	K	K	*K*	*K*
3ffc	I	J	44	44	*K*	*K*	K	K	*K*	*K*	K	K	*K*	*K*	K	K	*K*	*K*
3gsn	A	B	n/a	37	*n/a*	*K*	n/a	K	*n/a*	*K*	n/a	K	*n/a*	*F*	n/a	F	*n/a*	*F*
3h9s	D	E	37	37	*F*	*F*	F	F	*F*	*F*	F	F	*F*	*F*	F	F	*F*	*F*
3kpr	D	E	37	37	*K*	*K*	K	K	*K*	*K*	K	K	*K*	*K*	K	K	*K*	*K*
3kpr	I	J	37	37	*K*	*K*	K	K	*K*	*K*	K	K	*F*	*K*	F	K	*K*	*K*
3kps	D	E	37	37	*K*	*K*	K	K	*K*	*K*	K	K	*K*	*K*	K	K	*K*	*K*
3kxf	D	E	38	37	*K*	*K*	K	K	*K*	*K*	K	K	*K*	*K*	K	K	*K*	*K*
3kxf	M	O	38	37	*K*	*K*	K	K	*K*	*K*	K	K	*K*	*K*	K	K	*K*	*K*
3kxf	N	P	38	37	*K*	*K*	K	F	*F*	*K*	F	K	*F*	*K*	F	K	*F*	*K*
3mbe	C	D	44	44	*F*	*K*	K	K	*F*	*K*	F	K	*F*	*K*	F	K	*F*	*K*
3mbe	G	H	44	44	*F*	*K*	K	K	*F*	*K*	F	K	*F*	*K*	F	K	*F*	*K*
3mv8	D	E	44	44	*F*	*K*	F	K	*F*	*K*	F	K	*F*	*K*	F	K	*F*	*K*
3pwp	D	E	37	37	*K*	*K*	K	K	*K*	*K*	K	K	*K*	*K*	K	K	*K*	*K*
3qiu	C	D	n/a	37	*n/a*	*K*	n/a	K	*n/a*	*K*	n/a	K	*n/a*	*K*	n/a	K	*n/a*	*K*
3qiw	C	D	n/a	37	*n/a*	*K*	n/a	K	*n/a*	*F*	n/a	K	*n/a*	*K*	n/a	K	*n/a*	*K*

a) TCR chain pairs indicating the biological unit.
b) Residue IDs of the conserved glutamine residues at the center of rotation.
c) Flip state, after separate application of the tool to individual chains.
d) Flip state, after application of the tool to the complex.
e) Flip state before rigid body optimization.
f) Flip state, after separate application of the refinement to individual chains (Threshold 90°).
g) Flip state, after application of refinement tool to the complex (Threshold 90°).
Angular values of the refinements are listed in Additional file 8: Table S4
