# Supplementary material for: DynaDom: structure-based prediction of T cell receptor inter-domain and T cell receptor-peptide-MHC (class I) association angles
Source: BMC Struct Biol. 2017 Feb 2;17:2. doi: 10.1186/s12900-016-0071-7 (PMC5289058; doi:10.1186/s12900-016-0071-7)
Supplement: Supplementary file 8 — Table S4: Angular deviations with respect to the crystal structures after DynaDom glutamine refinement. (PDF 90 kb) [file 12900_2016_71_MOESM8_ESM.pdf]

DynaDom: Structure-based prediction of TCR inter-domain and TCR-pMHC association angles - T. Hoffmann, A. Marion, and I. Antes

Additional File 8

Table S4: Angular deviations with respect to the crystal structures after DynaDom glutamine refinement.

PDB	CH[a]		Angular Deviation[°]				PDB	CH[a]		Angular Deviation[°]			
			Single		Complex					Single		Complex	
ID	α	β	α	β	α	β	ID	α	β	α	β	α	β
1ao7	D	E	179.9	174.2	-168.3	-175.2	2vlr	I	J	11.4	7.9	7.7	5.7
1fo0	A	B	-179.5	179.9	-176.9	-176.0	3c5z	A	B	13.5	-5.8	9.3	2.5
1fyt	D	E	-11.5	-36.2	-135.3	-12.9	3c5z	E	F	13.7	-5.8	9.7	2.6
1j8h	D	E	174.2	-37.5	23.6	-10.5	3c60	A	B	-2.0	-18.5	96.0	-54.9
1kj2	A	B	-174.9	165.9	176.0	170.0	3c60	E	F	-3.2	-22.9	96.8	-56.9
1kj2	D	E	-161.2	167.8	-177.9	169.3	3c6l	A	B	33.5	157.2	69.2	121.4
1mi5	D	E	-168.6	5.6	8.0	6.4	3c6l	E	F	35.3	157.0	71.2	122.8
1mwa	A	B	-108.5	-178.1	176.1	-163.8	3d39	D	E	-154.7	175.1	-153.4	-177.4
1nam	A	B	179.9	158.8	168.1	174.0	3d3v	D	E	-165.6	174.2	-167.7	-172.0
1oga	D	E	7.8	16.8	3.4	14.8	3dxa	D	E	-147.1	17.7	-163.7	14.9
1qse	D	E	-173.3	157.0	-173.4	167.9	3dxa	I	J	-155.9	22.7	-172.6	19.9
1u3h	A	B	25.6	138.8	26.3	127.0	3dxa	N	O	-150.5	20.5	-162.9	12.7
1u3h	E	F	-2.2	134.1	-15.4	-149.7	3e2h	B	C	-2.8	-0.1	3.7	4.3
2bnq	D	E	-174.8	168.5	-176.5	-178.9	3e3q	C	F	5.4	-0.1	10.7	4.2
2bnr	D	E	-171.8	168.5	-173.5	-178.6	3e3q	d	e	2.3	-4.6	4.8	4.0
2e7l	A	D	20.9	157.0	24.3	162.1	3e3q	D	E	3.5	-3.7	7.1	2.8
2e7l	B	C	14.4	-4.3	22.1	-2.3	3e3q	I	J	5.7	-4.0	9.7	2.4
2esv	D	E	11.4	n/a	-1.5	n/a	3e3q	M	N	5.2	-0.1	10.5	3.9
2f53	D	E	0.8	-155.8	-9.0	-160.6	3e3q	R	S	1.2	-0.1	5.3	7.8
2f54	D	E	3.9	174.2	-20.9	-170.2	3e3q	V	W	3.7	-5.8	5.6	2.0
2f54	K	L	25.3	168.5	178.7	16.3	3e3q	Z	a	-5.8	0.2	-4.6	6.9
2gj6	D	E	-173.5	179.9	-172.7	-173.0	3ffc	D	E	-3.0	8.4	-0.5	19.2
2iam	C	D	-1.8	15.4	-9.0	17.4	3ffc	I	J	-5.2	17.2	-2.7	20.2
2ian	D	E	-5.8	14.8	-7.6	27.3	3gsn	A	B	n/a	154.8	n/a	157.7
2ian	I	J	-3.2	17.5	-3.7	18.8	3h9s	D	E	-180.0	174.2	-176.8	-177.8
2ian	N	O	-5.8	-5.8	-8.3	1.7	3kpr	D	E	-2.4	3.3	-7.3	1.4
2ian	S	T	-5.8	-5.8	4.1	-1.8	3kpr	I	J	180.0	-1.0	7.2	-2.7
2nx5	I	J	2.4	1.1	-10.9	8.2	3kps	D	E	12.8	-13.2	-7.8	-14.5
2nx5	N	P	21.1	11.4	12.9	7.3	3kxf	D	E	-2.0	-40.6	-5.1	-11.7
2nx5	T	U	10.5	16.4	-1.1	25.9	3kxf	M	O	-3.8	-44.2	3.4	-49.2
2oi9	B	C	5.3	-5.8	2.7	-3.5	3kxf	N	P	-166.3	-4.4	-166.0	14.7
2ol3	A	B	3.6	-11.5	10.9	-8.0	3mbe	C	D	180.0	-30.6	149.2	-43.1
2p5e	D	E	3.7	-157.6	-2.7	-159.2	3mbe	G	H	-180.0	-31.0	152.7	-38.2
2p5w	D	E	-3.0	-157.0	-14.9	-159.9	3mv8	D	E	-172.2	50.9	-174.7	22.9
2pxy	A	B	177.7	11.4	172.0	5.7	3pwp	D	E	5.6	2.3	3.7	9.4
2pye	D	E	-6.3	-157.0	-18.4	-160.4	3qiu	C	D	n/a	-0.2	n/a	14.1
2vlk	D	E	-8.8	23.1	-11.3	19.8	3qiw	C	D	n/a	4.2	n/a	-6.4
2vlr	D	E	3.7	9.4	0.8	7.6							

a) TCR chain ID contained in the same biological unit.
