# Supplementary material for: DynaDom: structure-based prediction of T cell receptor inter-domain and T cell receptor-peptide-MHC (class I) association angles
Source: BMC Struct Biol. 2017 Feb 2;17:2. doi: 10.1186/s12900-016-0071-7 (PMC5289058; doi:10.1186/s12900-016-0071-7)
Supplement: Supplementary file 9 — Figure S1: Discrimination of the models. (PDF 1267 kb) [file 12900_2016_71_MOESM9_ESM.pdf]

DynaDom: Structure-based prediction of TCR inter-domain and TCR-pMHC association angles - T. Hoffmann, A. Marion, and I. Antes
Additional File 9

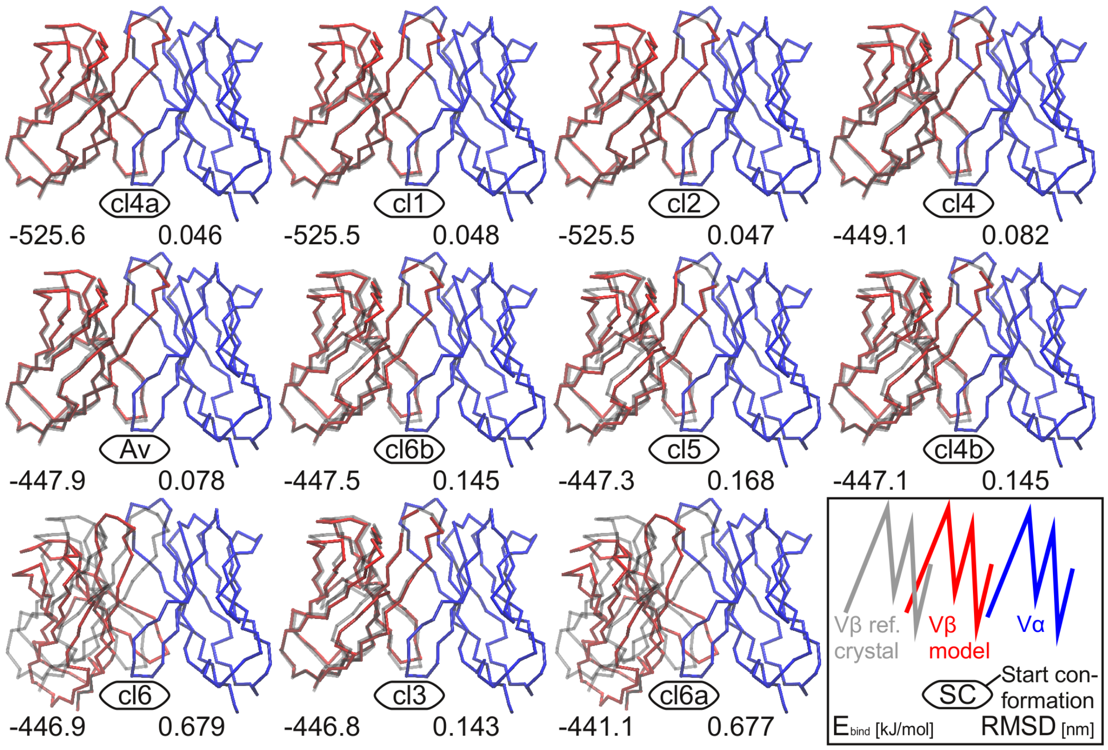

Figure S1: Discrimination of the models.

Eleven final models of structure 2p5e. Red: Vβ domain; blue: Vα domain; gray: crystal structure. Left side: interaction energy (kcal/mol); right side: RMSD (nm) (see also insertion above for explanation). The models are ordered according to their energy. Note that our method is able to discriminate accurate models of low RMSD with respect to the experimental structures (gray) based on the interaction energy between the Vα and the Vβ domains (first row). See Text S1 (Additional file 1) for the labeling of the 11 starting structures.
