# Supplementary material for: DynaDom: structure-based prediction of T cell receptor inter-domain and T cell receptor-peptide-MHC (class I) association angles
Source: BMC Struct Biol. 2017 Feb 2;17:2. doi: 10.1186/s12900-016-0071-7 (PMC5289058; doi:10.1186/s12900-016-0071-7)
Supplement: Supplementary file 10 — Figure S2: Influence of the restraint operator. (PDF 625 kb) [file 12900_2016_71_MOESM10_ESM.pdf]

DynaDom: Structure-based prediction of TCR inter-domain and TCR-pMHC association angles - T. Hoffmann, A. Marion, and I. Antes
Additional File 10

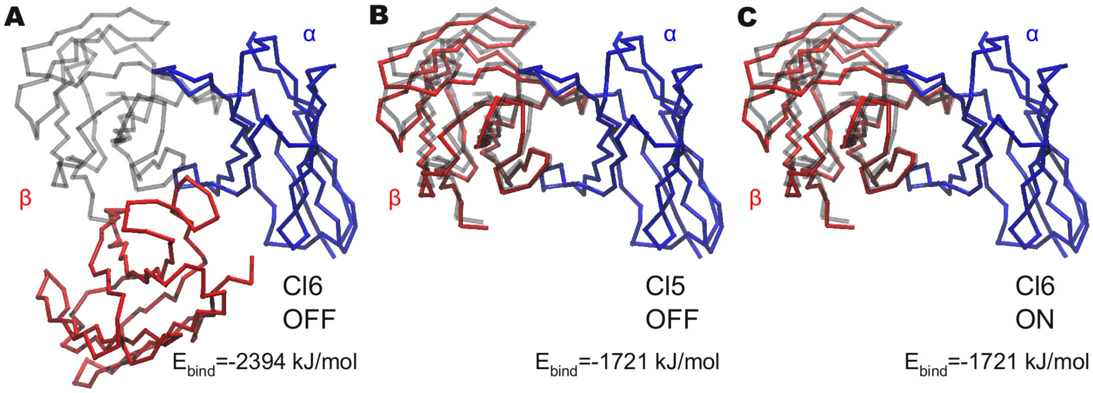

Figure S2: Influence of the restraint operator.
Final models of the structure 1kj2/DE after the optimization procedure (view: ligand binding site). The Vβ domain of the original experimental crystal structure is shown in transparent black as a reference. If the position of the Vβ domain is not constrained, the optimization from the starting conformation Cl6 leads to an unfavored model (A, RMSD 30.00 Å) with a better interaction energy than the preferred model starting from Cl5 (B, 1.54 Å), and thus the energy based prediction fails. C) By switching the restraints on, the model starting from Cl6 converges to the preferred conformation (RMSD 1.59 Å).
