# Supplementary material for: DynaDom: structure-based prediction of T cell receptor inter-domain and T cell receptor-peptide-MHC (class I) association angles
Source: BMC Struct Biol. 2017 Feb 2;17:2. doi: 10.1186/s12900-016-0071-7 (PMC5289058; doi:10.1186/s12900-016-0071-7)
Supplement: Supplementary file 11 — Table S5: pMHC optimization for the structure 1oga with different pMHC start conformations. (PDF 73 kb) [file 12900_2016_71_MOESM11_ESM.pdf]

DynaDom: Structure-based prediction of TCR inter-domain and TCR-pMHC association angles - T. Hoffmann, A. Marion, and I. Antes

Additional File 11

Table S5: pMHC optimization for the structure 1oga with different pMHC start conformations.

pMHC Start Conf. Angle[a]			E_{bind}	$RMSD_{C\alpha}$ [nm]		
x [°]	y [°]	z [°]	[kJ/mol]	Vβ	pMHC	Vβ&pMHC
-5.0	-5.0	-5.0	-1435.71	0.0273	0.9121	0.7260
-5.0	-5.0	0.0	-1466.90	0.0257	1.0704	0.8520
-5.0	-5.0	5.0	-1518.47	0.0312	1.1560	0.9202
-5.0	0.0	-5.0	-1240.59	0.0366	0.8944	0.7121
-5.0	0.0	0.0	-1510.11	0.0217	1.0945	0.8712
-5.0	0.0	5.0	-1526.07	0.0334	1.1955	0.9516
**-5.0**	**5.0**	**-5.0**	**-1839.48**	**0.0259**	**0.0259**	**0.0259**
**-5.0**	**5.0**	**0.0**	**-1838.86**	**0.0227**	**0.0303**	**0.0278**
-5.0	5.0	5.0	-1246.40	0.0241	0.9172	0.7301
0.0	-5.0	-5.0	-1710.15	0.0183	0.8772	0.6982
0.0	-5.0	0.0	-1427.67	0.0331	1.0375	0.8259
0.0	-5.0	5.0	-1250.48	0.0210	0.9642	0.7674
0.0	0.0	-5.0	-1245.79	0.0307	0.8868	0.7060
0.0	0.0	0.0	-1706.83	0.0208	0.9177	0.7304
0.0	0.0	5.0	-1251.17	0.0246	0.9829	0.7824
**0.0**	**5.0**	**-5.0**	**-1839.87**	**0.0248**	**0.0205**	**0.0222**
0.0	5.0	0.0	-1248.86	0.0369	0.9318	0.7419
0.0	5.0	5.0	-1584.93	0.0280	0.8707	0.6931
5.0	-5.0	-5.0	-1708.17	0.0277	0.8988	0.7155
5.0	-5.0	0.0	-1706.07	0.0202	0.9106	0.7248
5.0	-5.0	5.0	-1245.24	0.0251	1.0039	0.7991
5.0	0.0	-5.0	-1577.50	0.0356	0.5658	0.4508
**5.0**	**0.0**	**0.0**	**-1840.44**	**0.0160**	**0.0191**	**0.0180**
5.0	0.0	5.0	-1248.18	0.0219	1.0019	0.7975
5.0	5.0	-5.0	-1707.68	0.0283	0.9075	0.7224
**5.0**	**5.0**	**0.0**	**-1839.45**	**0.0251**	**0.0229**	**0.0237**
5.0	5.0	5.0	-1708.90	0.0264	0.9007	0.7170

a) The TCR conformation is set to the crystal structure. The pMHC deviates from the zero-conformations by the values x, y, and z (Euler angle components).
